# DeepCCDS: Interpretable Deep Learning Framework for Predicting Cancer Cell Drug Sensitivity through Characterizing Cancer Driver Signals

**DOI:** 10.1002/advs.202416958

**Published:** 2025-05-21

**Authors:** Jiashuo Wu, Jiyin Lai, Xilong Zhao, Ziyi Wang, Yongbao Zhang, Liqiang Wang, Yinchun Su, Yalan He, Siyuan Li, Ying Jiang, Junwei Han

**Affiliations:** ^1^ College of Bioinformatics Science and Technology Harbin Medical University Harbin 150081 China; ^2^ College of Basic Medical Science Heilongjiang University of Chinese Medicine Harbin 150040 China

**Keywords:** deep learning, drug sensitivity, feature representation, precision oncology, self‐supervised neural network

## Abstract

Accurate characterization of cellular states is the foundation for precise prediction of drug sensitivity in cancer cell lines, which in turn is fundamental to realizing precision oncology. However, current deep learning approaches have limitations in characterizing cellular states. They rely solely on isolated genetic markers, overlooking the complex regulatory networks and cellular mechanisms that underlie drug responses. To address this limitation, this work proposes DeepCCDS, a Deep learning framework for Cancer Cell Drug Sensitivity prediction through Characterizing Cancer Driver Signals. DeepCCDS incorporates a prior knowledge network to characterize cancer driver signals, building upon the self‐supervised neural network framework. The signals can reflect key mechanisms influencing cancer cell development and drug response, enhancing the model's predictive performance and interpretability. DeepCCDS has demonstrated superior performance in predicting drug sensitivity compared to previous state‐of‐the‐art approaches across multiple datasets. Benefiting from integrating prior knowledge, DeepCCDS exhibits powerful feature representation capabilities and interpretability. Based on these feature representations, we have identified embedding features that could potentially be used for drug screening in new indications. Further, this work demonstrates the applicability of DeepCCDS on solid tumor samples from The Cancer Genome Atlas. This work believes integrating DeepCCDS into clinical decision‐making processes can potentially improve the selection of personalized treatment strategies for cancer patients.

## Graphical abstract

DeepCCDS leverages prior knowledge and self‐supervised learning to model cancer driver signals for drug sensitivity prediction. It captures complex regulatory patterns enabling more biologically informed representations. The framework outperforms existing methods across datasets, offering improved accuracy and interpretability. It enables discovery of informative features for drug screening and demonstrates potential for guiding personalized treatment decisions in solid tumors.

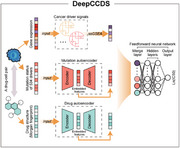

## 1 Introduction

Cancer is a highly heterogeneous disease, displaying a range of genetic diversity and phenotypic variability.^[^ ^1^ ^]^ Annually, numerous novel treatment modalities are subjected to clinical evaluation for their efficacy against diverse cancer types, yet less than 4% receive approval from the US Food and Drug Administration.^[^ ^2^ ^]^ Even with favorable outcomes post‐therapy, the considerable heterogeneity may lead to eventual tumor progression.^[^ ^3^ ^]^ These challenges highlight the need for more sophisticated approaches to predict individual responses to cancer treatments.

Genomic and transcriptomic characteristics have been proven to correlate significantly with patients’ responses to cancer treatments.^[^ ^4^, ^5^ ^]^ Owing to the limited availability of large cancer patient cohorts, large‐scale cell line assays and comprehensive multi‐omics databases like GDSC^[^ ^6^ ^]^ and CCLE^[^ ^7^ ^]^ have been instrumental in characterizing biological heterogeneity and enhancing our understanding of drug response mechanisms. Recently, many computational approaches based on deep learning and large‐scale cell line assays have been developed to improve drug sensitivity prediction. Despite achieving promising predictive results, existing approaches remain controversial in terms of characterizing cell features. For example, liu et al. introduced the tCNNS, which predicts the drug sensitivity of cell lines by utilizing genetic variation based on convolutional neural networks.^[^ ^8^ ^]^ Jiang et al. developed DeepTTA, a deep learning model that integrates a transformer architecture and a neural network for predicting the anti‐cancer drug sensitivity of cell lines using gene expression data.^[^ ^9^ ^]^ Chiu et al. introduce DeepDR for predicting the efficacy of cancer treatments using a deep learning model, which uses gene mutation and expression profiles of cancer cell lines.^[^ ^10^ ^]^ These approaches use genomic and/or transcriptomic features of all genes to characterize cells, which may incorporate inherent noise and increase the complexity of the model. Therefore, training models using more insightful features, such as some important biomedical entity, appears to be a superior strategy. For example, Precily presented by Chawla et al. leverages the pathways instead of genes to predict anti‐cancer drug sensitivity.^[^ ^1^ ^]^ Chang et al. developed CDRscan, which can infer drug sensitivity using only the mutation status of cancer driver genes.^[^ ^11^ ^]^ Driver genes play a central role in tumor progression, and their genetic status may be directly related to the cellular response to drugs.^[^ ^12^, ^13^ ^]^ CDRscan successfully improves the model's predictive ability by using driver genes to characterize cellular states. However, the approach solely considers the genetic state of driver genes, overlooking the broader cellular perturbations and their impact on drug sensitivity. Cancer cell response to drugs is governed by the interplay of multiple signaling cascades, rather than the isolated genetic profile of single genes.^[^ ^14^ ^]^ Therefore, we believe incorporating the complex perturbation of cancer driver genes would further enhance the model's predictive accuracy.

Here, we developed DeepCCDS, a deep learning framework for cancer cell drug sensitivity prediction through characterizing cancer driver signals. This provides a more nuanced understanding of drug response mechanisms, potentially leading to more accurate predictions of drug sensitivity. Specifically, this framework consists of four main components: **(1)** A prior knowledge network is used to characterize signal transduction of driver genes as biological pathways. The activities of these pathways serve as an embedded representation of the cell's gene expression profile; **(2)** A mutation autoencoder is used to learn embedded representations of the mutational states of driver genes; **(3)** A drug autoencoder is used to learn embedded representations of drug molecular structures; **(4)** A feedforward neural network is used to integrate these three embedded features and predict the sensitivity value of cells to drugs. DeepCCDS demonstrates excellent predictive capability in terms of regression‐based and classification‐based metrics in different datasets, specific cells, or specific drugs, outperforming some well‐cited deep learning‐based approaches and traditional machine learning models. Through systematic model interpretation, we demonstrate that DeepCCDS can effectively abstract original features and discover new features related to drug response. We also applied DeepCCDS to clinical patient cohorts of The Cancer Genome Atlas (TCGA) to determine the potential for extrapolating this approach in precision oncology. Overall, DeepCCDS effectively enhances the accuracy and interpretability of current drug sensitivity prediction approaches and shows promise in advancing personalized medicine.

## 2 Results

### 2.1 Characterization of Cancer Driver Signals for Drug Sensitivity Prediction

**Figure 1:**
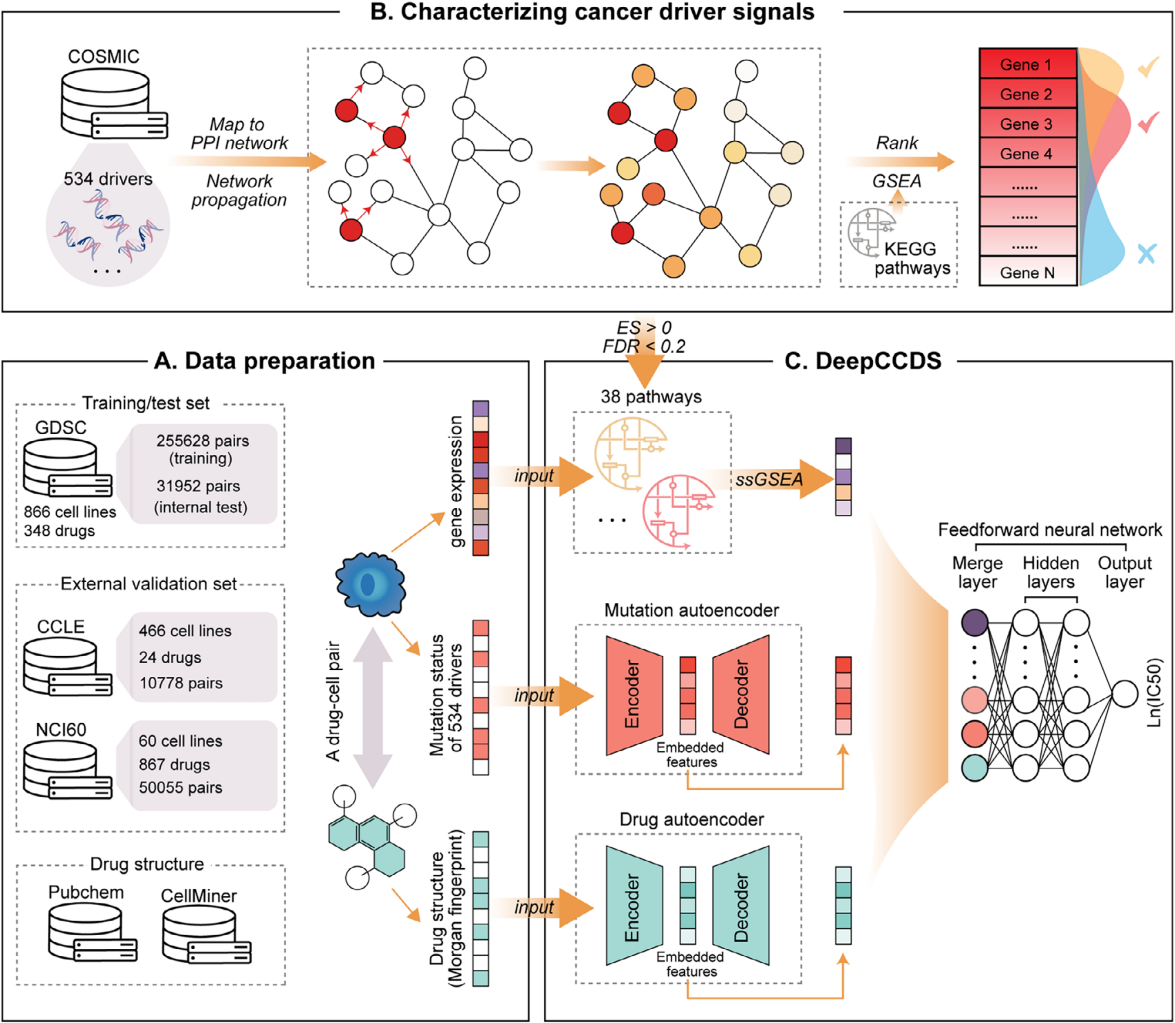
The schematic overview of DeepCCDS.

**Figure 2:**
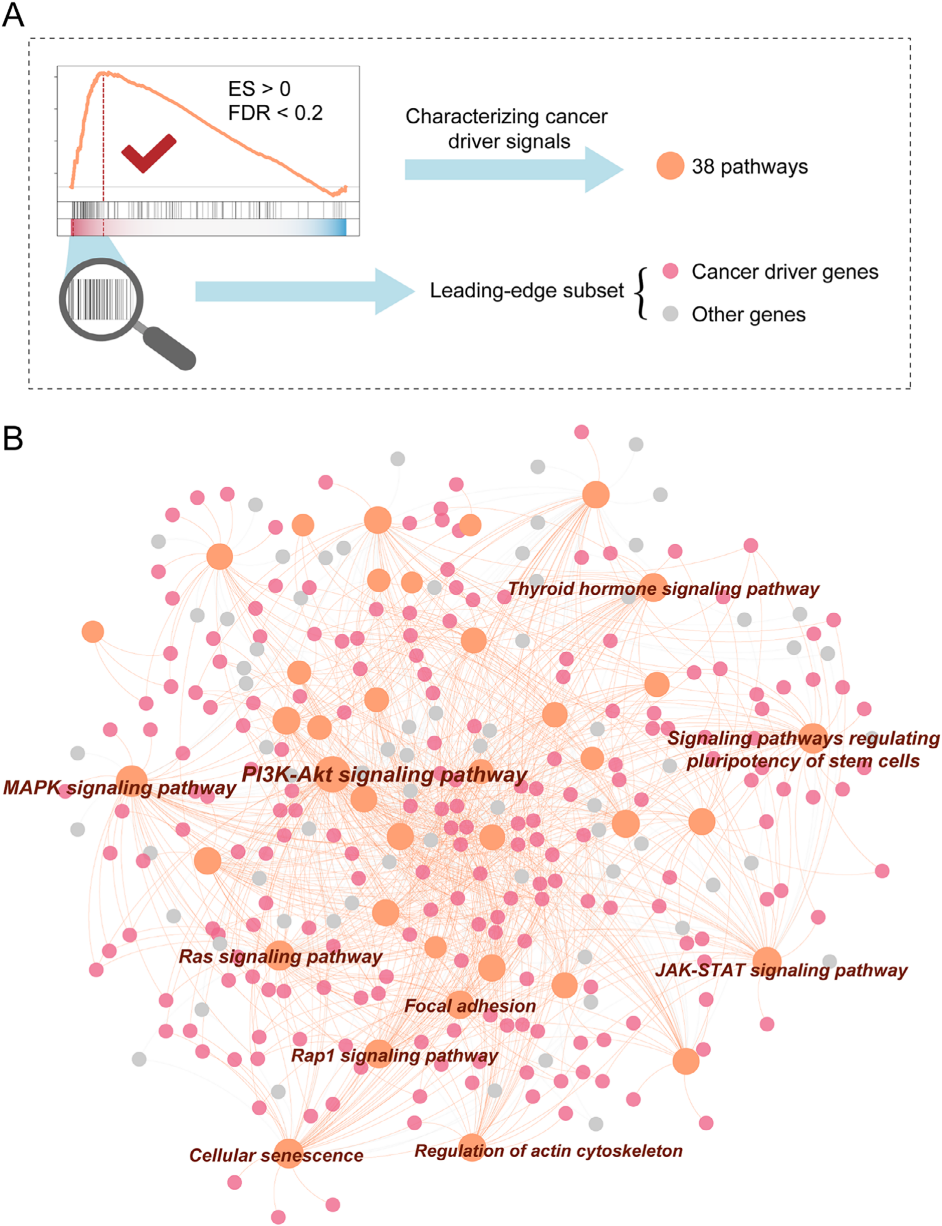
Characterizing cancer driver signals as biological pathways. A) 38 pathways were determined based on enrichment analysis thresholds (ES > 0, FDR < 0.2) to characterize cancer driver signals. The leading‐edge subset includes cancer driver genes and other genes influenced by driver genes. B) A pathway‐gene relationship network, where each pathway is connected to its corresponding leading‐edge subset genes. The size of a pathway node indicates its degree, with larger nodes representing higher degrees.

DeepCCDS is a novel computational framework for predicting the sensitivity of specified cells to drugs (**Figure** **1**). This framework integrates the prior knowledge network to characterize the key signals in cancer cell development and proliferation. Considering these factors can effectively improve the accuracy of prediction for drug sensitivity. Specifically, we characterized the cancer driver signals as 38 pathways through enrichment analysis (see Methods section; **Figure** **2**A; Table S1, Supporting Information). The GSEA results' leading‐edge subset represents key genes driving the enrichment signal within the gene set.^[^ ^15^ ^]^ We found a higher proportion of driver genes in the leading‐edge subset of each pathway (Figure 2A,B). This indicates that these pathways can effectively reflect the action mechanisms of driver genes. We observed many well‐studied cancer development‐related key pathways, such as the MAPK signaling pathway,^[^ ^16^ ^]^ PI3K‐Akt signaling pathway,^[^ ^17^ ^]^ and JAK‐STAT signaling pathway.^[^ ^18^ ^]^ Moreover, these pathways have also been proven to be associated with cancer treatment response and are used as therapeutic targets for some cancers in clinical settings.^[^ ^19^, ^20^, ^21^ ^]^ The single sample gene set enrichment analysis (ssGSEA) algorithm was then applied to calculate the activities of the 38 pathways. Using the pathway activity instead of high‐dimensional gene expression data can simplify the complexity of the model while retaining key biological information and reflecting the biological mechanisms of driver genes.

### 2.2 Comprehensive Evaluation for the DeepCCDS Model

**Figure 3:**
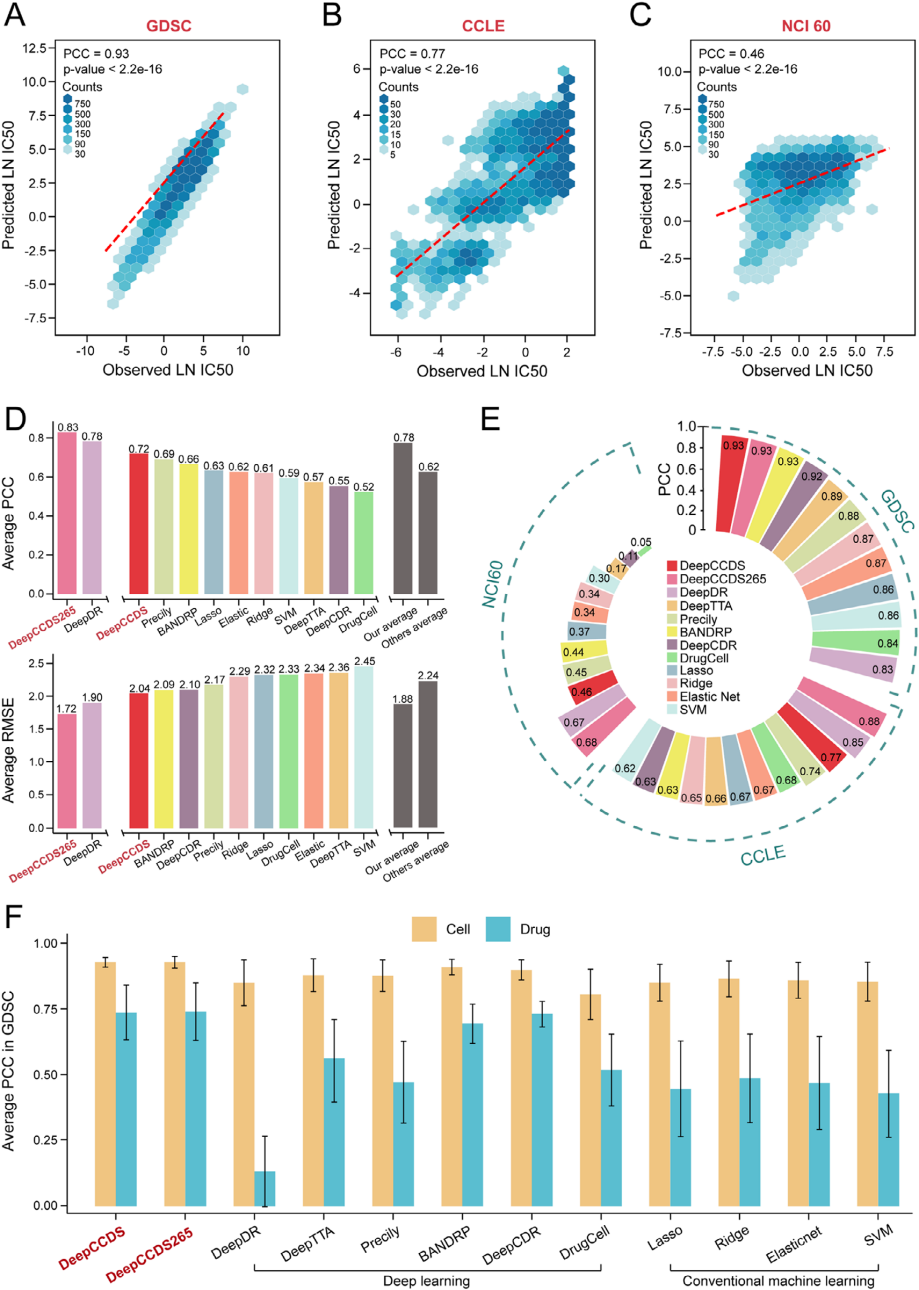
Comprehensive performance evaluations of DeepCCDS. A–C) The correlation between predicted LN IC50 by DeepCCDS and observed LN IC50 across different datasets: (A) GDSC, (B) CCLE, and (C) NCI 60. D) Comparison of overall performance (average PCC or RMSE) in different approaches across three datasets. “Our average” refers to the mean overall performance of DeepCCDS and DeepCCDS265, while “Other average” refers to the mean overall performance of the other methods. E) The detailed PCC of different approaches in three datasets. F) Comparison of different approaches, showing the mean (bars) and standard deviation (error bars) of prediction performance for each cell line across all drugs and for each drug across all cell lines.

To execute the training process, we obtained a total of 319 543 drug‐cancer cell line pairs and their corresponding sensitivity data from the GDSC database. All samples (cell‐drug pairs) were randomly divided into training (80%), validation (10%), and test (10%) sets. We determined the structure of the autoencoders and the training parameters for the complete DeepCCDS through pre‐training (see Methods section). By comparing various parameter combinations, the structure that achieved the lowest BCE for both drug and mutation autoencoders was identified as having two hidden layers with 300 and 100 neurons, respectively, and a bottleneck layer with 30 neurons (Figure S1, Supporting Information). Comparing the predicted and observed drug sensitivity according to Pearson correlation coefficient (PCC) and Root Mean Square Error (RMSE), the optimal learning rate for complete training was determined as 1e‐3, with a batch size of 1024 (Figure S2, Supporting Information). Finally, using the predetermined optimal parameters, DeepCCDS was retrained on the training set. The obtained trained model demonstrated highly accurate prediction in the test set (PCC = 0.93, p‐value < 2.2e‐16; **Figure** **3**A). To demonstrate the predictive robustness of DeepCCDS, we performed a 10‐times Monte Carlo cross‐validation on all samples from the GDSC dataset, using an 8:1:1 ratio for training, validation, and test sets. We then performed model training and tests on these newly partitioned sets 10 times. We found that the natural logarithm of the half‐maximal inhibitory concentration (LN IC50) predicted from these ten models exhibited strong and consistent correlations with the observed values (PCC = 0.93, p‐value < 2.2e‐16; Figure S3, Supporting Information), demonstrating the robustness of DeepCCDS. To verify the generalizability of DeepCCDS, we selected two external validation sets (CCLE and NCI60) to assess its predictive performance. In CCLE, the drug sensitivity predicted by DeepCCDS showed high consistency with observed LN IC50 (PCC = 0.77, p‐value < 2.2e‐16; Figure 3B). Despite the NCI60 dataset using GI50 to reflect cell growth inhibition, we could still confirm the overall consistency of DeepCCDS predictions (PCC = 0.46, p‐value < 2.2e‐16; Figure 3C). The ability of DeepCCDS to predict across these two metrics demonstrates its capacity to capture general characteristics of drug efficacy.

We then conducted ablation experiments to analyze the influence of different features on model performance. Specifically, we designed three variants of our method by training the model with different feature sets. The first variant was trained using only gene mutation data. The second variant was trained using only pathway activity data. The third variant was trained using a combination of gene mutation and gene expression data. We trained each variant and evaluated their predictive performance on both internal and external validation datasets. Here, we employed both regression‐based and classification‐based evaluation strategies to assess the model performance (see the Methods section). We found that all three variants led to decreased predictive performance compared to the original DeepCCDS model, with the most significant drop observed when using only mutation data (Figure S4, Supporting Information). This is likely because mutation status, typically encoded as binary values, fails to reflect the functional heterogeneity of different mutations and cannot capture their downstream regulatory consequences. In contrast, DeepCCDS integrates driver mutations with their regulated pathways, effectively combining the source of cancer signals with their functional impact. The superior performance of DeepCCDS across both internal and external validation datasets underscores the importance of this integrative feature strategy in accurately modeling cancer drug response.

We conducted benchmark tests comparing DeepCCDS with several well‐cited deep learning frameworks (DeepTTA,^[^ ^9^ ^]^ DeepDR,^[^ ^10^ ^]^ Precily,^[^ ^1^ ^]^ BANDRP,^[^ ^22^ ^]^ DeepCDR^[^ ^23^ ^]^ and DrugCell^[^ ^24^ ^]^) and traditional machine learning algorithms (lasso, ridge, elastic net regression models, and SVM). These deep learning‐based frameworks use genomic (DrugCell), transcriptomic (DeepTTA, Precily), or multi‐omics features (DeepDR, BANDRP, DeepCDR) to characterize cell lines, which is also a common usage in existing methods. The DeepCCDS framework designed here not only integrates different omics information but also considers the perturbation of driver signals on transcription. This multi‐level integration can capture more detailed and accurate cell‐drug response information. Here, we compare the predictive performance of DeepCCDS with these outstanding computational frameworks across different datasets. It is important to note that the DeepDR method can only predict cell sensitivity to a specific set of 265 drugs. To ensure a fair comparison, we defined DeepCCDS265 as the results of DeepCCDS predicting cell sensitivity to these 265 drugs, specifically for comparison with DeepDR. We first compared the overall performance of DeepCCDS with other approaches across the GDSC, CCLE, and NCI60 datasets. The results demonstrate that our approach achieved superior average performance across the three datasets. Specifically, our approach achieved a 25% improvement in PCC and a 16% reduction in RMSE compared to other approaches (Figure 3D, “Our average” versus “Others average”). In terms of classification performance, compared to other approaches, our approach achieved improvements of 11% and 30% in the Area Under the Receiver Operating Characteristic Curve (AUROC) and F1 scores (Figure S5A,B, Supporting Information, “Our average” versus “Others average”), respectively. For each evaluation metric, our approach consistently demonstrates a substantial advantage over the other 10 approaches across three datasets (Figure 3E and Figure S5C,E, Supporting Information).

Next, we assessed the application of different approaches in specific cells or drugs. We calculated the PCC, RMSE, AUROC and F1 score between predicted and observed response for each cell across all drugs, then computed the mean and standard deviation of the four metrics for all cells, respectively. The same process was applied to each drug. Comparisons across different datasets consistently demonstrated that DeepCCDS achieved the best performance compared to other approaches for specific cells or drugs (Figure 3F and Figures S6–S8, Supporting Information). In conclusion, comprehensive model evaluation has demonstrated the superior performance of DeepCCDS over other approaches in multiple aspects.

Previously, we trained and compared the models using the “mix split” strategy, where the training, validation, and test sets were randomly divided. To enable a more comprehensive comparison with existing models, we incorporated three additional data‐splitting strategies: “cell line split,” “drug split,” and “both split.” The “cell line split” ensures that cell lines in the training, validation, and test sets do not overlap, allowing us to assess the model's ability to generalize to completely unseen cell lines. Similarly, the “drug split” ensures that no drugs overlap across the datasets, evaluating the model's performance in predicting new drugs. The “both split” strategy is the most stringent, ensuring that cell line–drug pairs are entirely non‐overlapping across the training, validation, and test sets, thus testing the model's ability to predict novel cell line–drug pairs in a real‐world setting. We trained our model under these different split strategies and evaluated its predictive performance across various datasets, comparing it against other methods. Under various data splitting strategies, our method achieved competitive performance. Especially in the external validation cohort, our method consistently outperformed all other approaches in different evaluation metrics (Figures S9–S11, Supporting Information). These results indicate the robustness and strong generalizability of our method across diverse experimental settings.

In addition to performance, we also evaluated and compared the computational efficiency of different deep learning methods. To ensure a fair comparison, we trained these models under the same hardware environment (NVIDIA GeForce RTX 4090 GPU and 128GB of RAM) and software environment (Python 3.8 and Pytorch 2.1.0). The DeepCDR method was excluded from the comparison because it was trained using an older version of Python and the “TensorFlow” environment. The results show that our method has significantly lower runtime and memory usage compared to most other deep learning‐based approaches (Figure S12, Supporting Information).

### 2.3 High‐Quality Feature Representation

**Figure 4:**
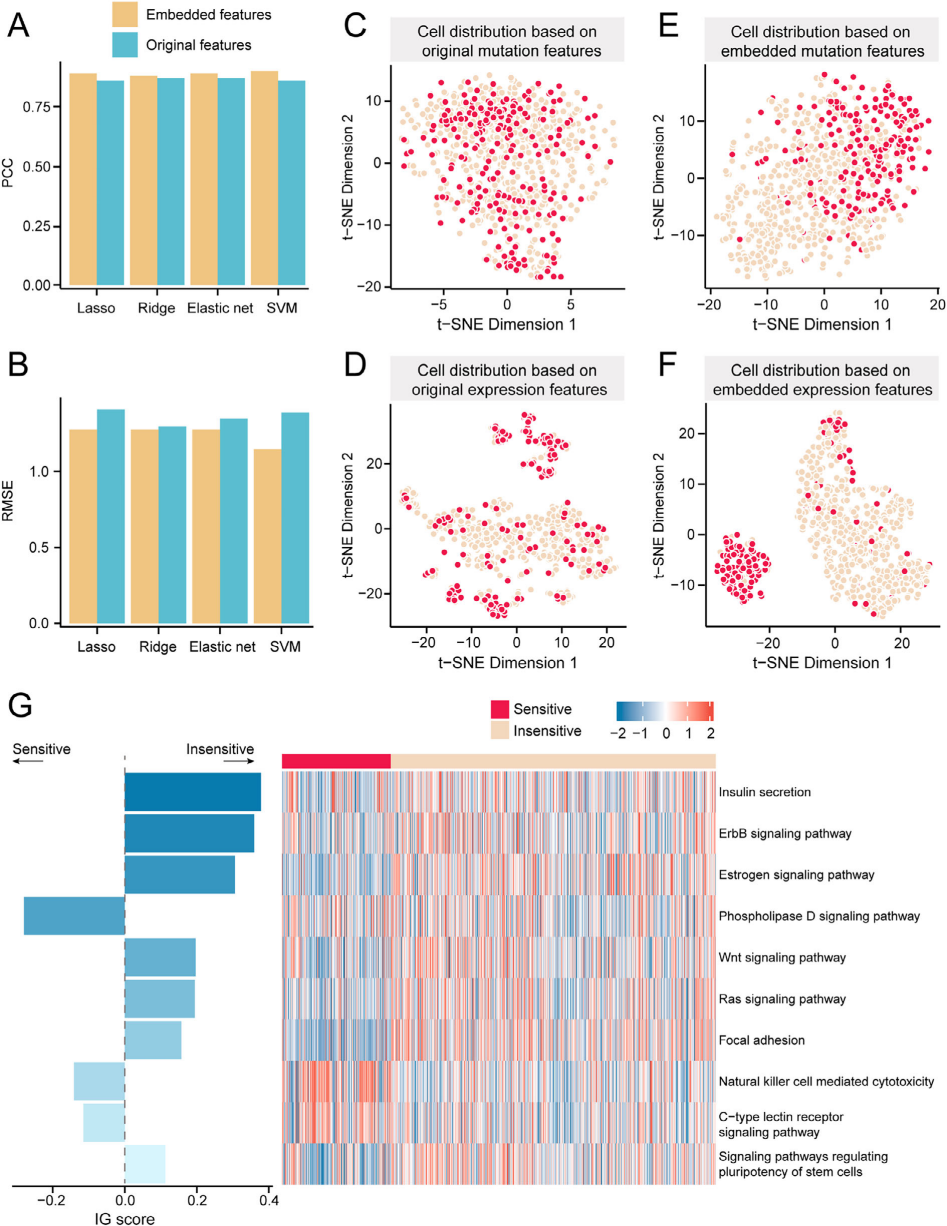
Analysis of embedded features generated by DeepCCDS. A,B) Comparison of (A) PCC and (B) RSEM of drug sensitivity prediction between machine learning models trained on original and embedded features. C–F) Cell distribution based on t‐SNE algorithm. The cell distribution is respectively based on original mutation features (C), original expression features (D), embedded mutation features (E), and embedded expression features (F). The cells were divided into sensitive and insensitive groups based on the quartiles of average sensitivity. G) The IG scores and activity heatmap of the top 10 pathways that are most important for the prediction of drug sensitivity.

Our previous results indicated that traditional machine learning methods demonstrated weaker predictive capabilities compared to deep learning approaches, particularly in the internal validation set. This may be attributed to the high signal‐to‐noise ratio resulting from the high dimensionality of input features.^[^ ^25^ ^]^ Upon completing DeepCCDS training, the model acquired the ability to learn the embedded features of cells and drugs. Thus, the original mutations and drug structures were represented by 30 embedded mutation and drug features for each drug‐cell pair based on the mutation and drug structure autoencoders, respectively, and the original expression features were represented by 38 pathway activities (called embedded expression features). Consequently, we trained machine learning models using these embedded features. We observed that models trained on embedded features exhibited superior predictive performance (higher PCC and lower RMSE) compared to those trained on original features (**Figure** **4**A,B). Subsequently, we calculated the average sensitivity of each cell to all drugs and categorized cells into sensitive and insensitive groups based on the first quartile of this average sensitivity. We utilized the t‐SNE algorithm to visualize cell distribution based on both original and embedded features. We observed low separation between the sensitive and insensitive groups in the 2D space of original features (Figure 4C,D). In contrast, the two cell groups were well‐differentiated in the 2D space of embedded features (Figure 4E,F). Moreover, Figure 4E,F reveals that expression embedding features (38 pathways) provide clearer cell localization compared to mutation embedding features. This further suggests that integrating the cancer driver signals can effectively enhance the characterization of cellular states. In summary, these results demonstrate that DeepCCDS can generate high‐quality feature representations.

### 2.4 Analysis of Embedded Feature Importance through Model Interpretation

#### 2.4.1 Gene Expression Embedding Features

We conducted a feature importance analysis to further understand the relationship between the high‐quality features learned by DeepCCDS and drug sensitivity. First, using the IG method,^[^ ^26^ ^]^ we calculated the IG score for each dimension (pathway) of the gene expression embedding vector (see Methods section). We ranked the 38 pathways based on the absolute IG scores and presented the top ten. As shown in Figure 4G, the Insulin secretion pathway demonstrates the highest positive contribution to predicting LN IC50, suggesting it may reduce drug sensitivity. Previous studies have shown that the Insulin secretion pathway is abnormally expressed in certain cancer types, and this abnormality may lead to drug resistance.^[^ ^27^ ^]^ Other top positive pathways, such as the ErbB signaling pathway and Estrogen signaling pathway, have also been proven to be associated with resistance mechanisms in previous research.^[^ ^28^, ^29^ ^]^ These pathways exhibit higher activity in the insensitive group (Figure S13A–C, Supporting Information). Pathways with negative contributions, such as Natural killer cell‐mediated cytotoxicity, have been shown to enhance the effectiveness of cancer chemotherapy.^[^ ^30^ ^]^ This pathway demonstrates significantly higher activity in the sensitive group (Figure S13D, Supporting Information).

#### 2.4.2 Gene Mutation Embedding Features

**Figure 5:**
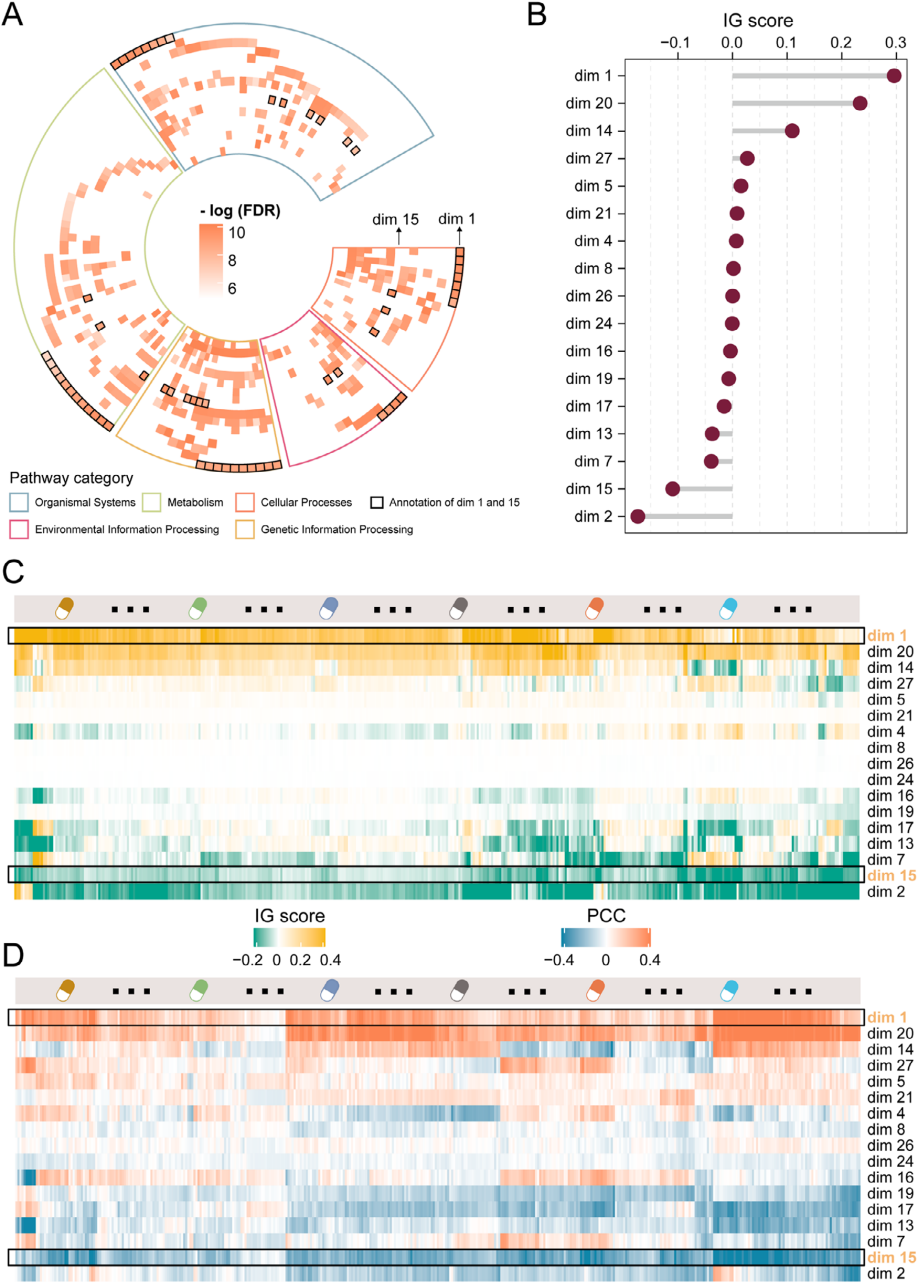
Biological significance of mutation embedding features. A) Biological annotation of mutation embedding features. Different colored regions represent different annotation pathway categories. Annotations for dimensions 1 and 15 are highlighted with bold borders. Detailed annotation names are shown in Figure S9, Supporting Information. B) The importance (IG score) of biologically annotated feature dimensions to drug sensitivity prediction. C) Heatmap of the importance (IG scores) for each dimension associated with sensitivity of specific drugs. D) Heatmap of the correlation (PCC) between feature values across different dimensions and drug sensitivity of cells.

The mutation embedding features are 30D vectors from the encoder network of mutation autoencoder. Each dimension of the vector lacks inherent biological meaning and cannot directly reflect specific biological mechanisms. To deepen our understanding of these non‐biological entities, we first performed pathway annotation for each dimension (see Methods section). We annotated biological pathways for each embedding dimension based on enrichment analysis with FDR < 0.05. A total of 17 dimensions were successfully annotated. We discovered that each dimension possesses unique pathway annotations (**Figures** **5**A and S14, Supporting Information). Notably, dimensions 1 and 15 (hereafter called dim 1 and 15) shared only two biological mechanisms. This phenomenon indicates that the autoencoder in DeepCCDS can effectively capture the main variations in the original data, reflecting more comprehensive biological information in a low‐dimensional representation, and contributing to improved predictive power for drug sensitivity. Moreover, we found that these two dimensions showed overall opposite directions of correlation with different pathway categories (Figures S15 and S16, Supporting Information). Pathways in the “Cellular Processes” and “Genetic information processing” categories have been confirmed by multiple studies to potentially lead to drug resistance in cancer cells.^[^ ^31^, ^32^, ^33^, ^34^ ^]^ For example, the cell cycle pathway belonging to the “Cellular Processes” category can lead to drug resistance by disrupting checkpoint functions and inducing cells to enter a quiescent state.^[^ ^35^ ^]^ The DNA replication pathway in the “Genetic information processing” category can lead to drug resistance by enhancing DNA repair mechanisms, allowing cancer cells to fix DNA damage caused by chemotherapy, thereby evading cell death​.^[^ ^36^, ^37^ ^]^ Both pathways are positively correlated with dim 1. The phagosome pathway in the “Cellular Processes” category can induce drug resistance by using tumor‐associated macrophages to create an immunosuppressive environment and reduce drug effectiveness.^[^ ^38^ ^]^ Proteasome pathways in the “Genetic information processing” category contribute to drug resistance by maintaining protein homeostasis in cancer cells.^[^ ^39^ ^]^ Both pathways are negatively correlated with dim 15. These suggest that dims 1 and 15 may influence cancer drug responses in opposite directions (inhibition or promotion).

Next, we analyzed the importance of these features on drug sensitivity. As shown in Figure 5B, we calculated and displayed the IG scores for the 17 dimensions. Interestingly, we observed that dims 1 and 15 had substantial importance. We then calculated the importance of 17 dimensions for sensitivity to specific drugs. Dim 1 consistently showed positive contributions to predicting sensitivity for all drugs, while dim 15 showed negative contributions (Figure 5C). We then calculated the PCC between the feature values of each dimension in cells and the LN IC50 values of each drug acting on the cells (Figure 5D). Since lower IC50 values indicate higher sensitivity to drugs, negative correlations in the figure suggest that the feature dimension promotes drug response, while positive correlations indicate that the dimension inhibits drug response. The results of importance and correlation analyses are consistent with the above findings, which show that dims 1 and 15 inhibit and promote cancer cell drug sensitivity, respectively.

**Figure 6:**
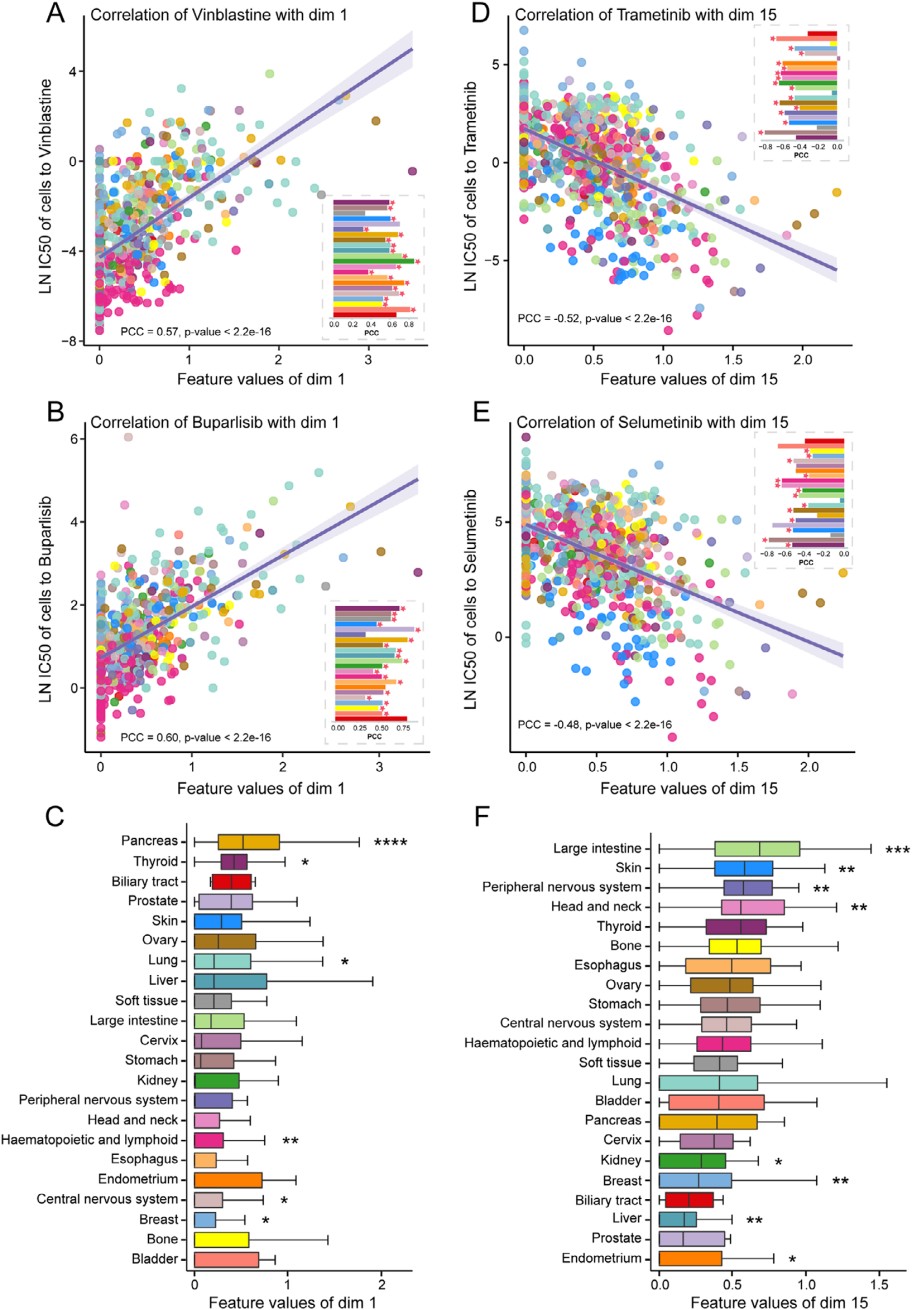
Correlation analysis of dimensions 1 and 15 with the specific drugs. A) The correlation between the feature values of dimension 1 and LN IC50 of cells to Vinblastine. The bar charts inside the scatter plot represent correlations within specific cell types. The red stars indicate a significant correlation between feature values in particular cell types and drug sensitivity. B) The correlation between the feature values of dimension 1 and LN IC50 of cells to Buparlisib. C) Comparison of feature values of dimension 1 across different cell types. We used a two‐sided Wilcoxon rank‐sum test to assess the differences between each group and all other patients (**** *p* < 1e‐4; *** 1e‐4 < *p* < 1e‐3; ** 1e‐3 < *p* < 1e‐2; * 1e‐2 < *p* < 5e‐2). D) The correlation between the feature values of dimension 15 and LN IC50 of cells to Trametinib. E) The correlation between the feature values of dimension 15 and LN IC50 of cells to Selumetinib. F) Comparison of feature values of dimension 15 across different cell types.

Among these drugs, Vinblastine and Buparlisib showed the strongest correlations with dim 1 (PCC = 0.57 and 0.60, p‐value < 2.2e‐16; **Figure** **6**A,B; Table S2, Supporting Information). Specifically, the sensitivity of almost every cell type to these two drugs showed significant positive correlations with feature values of dim 1. This suggests that dim 1 may have a strong inhibitory effect on the efficacy of these two drugs, with cells having lower feature values of dim 1 being more likely to respond to these drugs. Through comparison, we found that dim 1 has significantly lower feature values in the “breast,” “central nervous system,” and “haematopoietic and lymphoid” cell lines (Figure 6C). Notably, Vinblastine and Buparlisib have been approved for treating breast cancer, neuroblastoma, and various lymphomas. Similarly, we analyzed Trametinib and Selumetinib, which showed the strongest negative correlations with dim 15 (PCC = −0.52 and −0.48, *p*‐value < 2.2e‐16; Figure 6D,E; Table S3, Supporting Information). This implies that cell types with higher feature values in dim 15 may be more sensitive to these two drugs. Dim 15 has significantly higher feature values in “large Intestine,” “skin,” “peripheral nervous system,” and “head and neck” cell lines (Figure 6F). We also confirmed through DrugBank that Trametinib has been approved for treating colorectal cancer and melanoma, while Selumetinib is used for treating neurofibromatosis. In conclusion, through model interpretation, we revealed the relationship between embedded features and cell sensitivity to drugs. Dims 1 and 15 have the potential to be used independently for predicting and characterizing drug sensitivity and may aid in screening for new drug indications.

### 2.5 Predicting Patient Clinical Response

**Figure 7:**
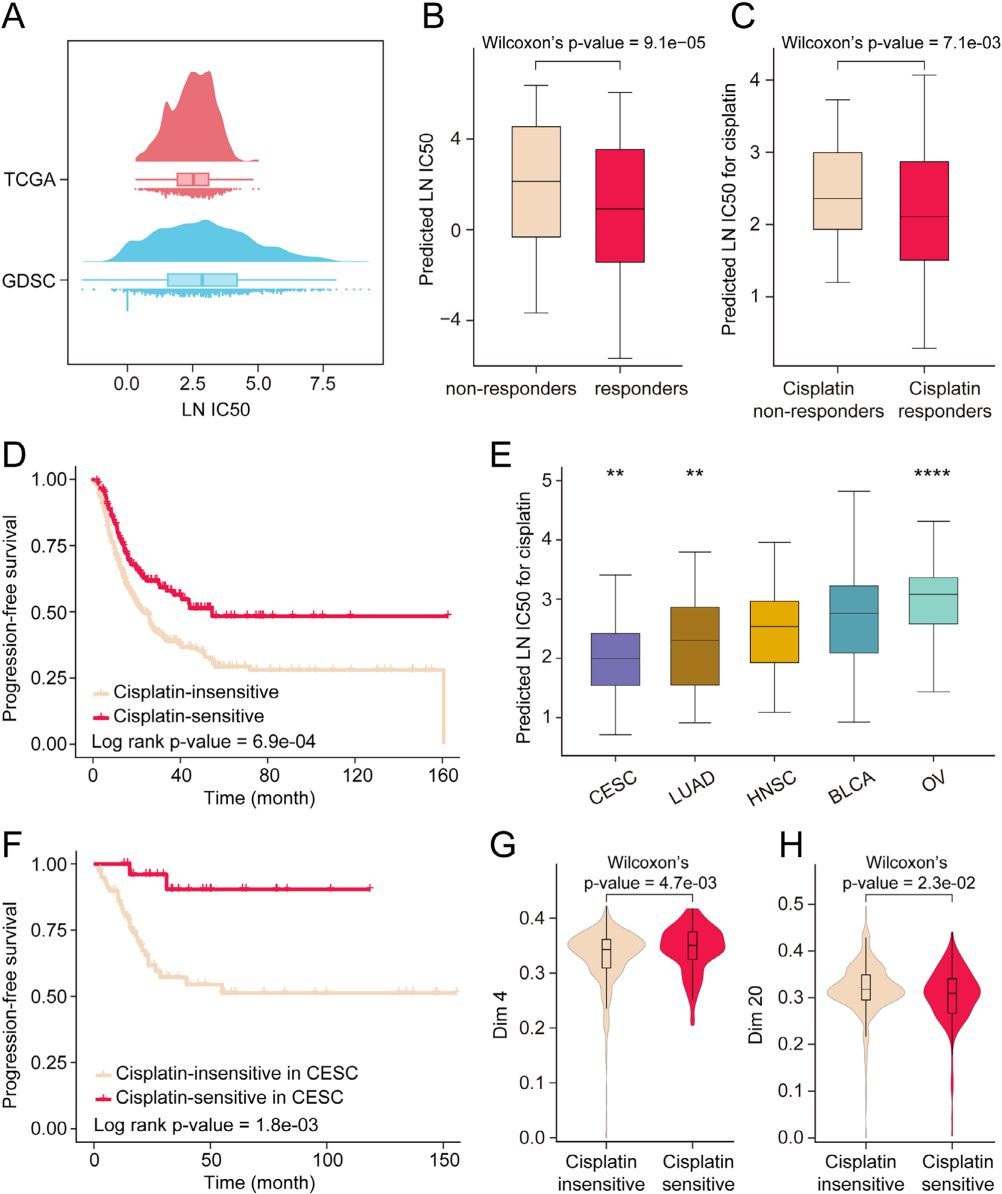
Application of DeepCCDS in solid tumor samples. A) Comparing the LN IC50 distributions of cell lines in GDSC to 25 drugs with the predicted sensitivity distributions of TCGA patients to the same 25 drugs. B) Comparing the predicted LN IC50 between all responders and non‐responders. C) Comparing the predicted LN IC50 between all responders and non‐responders of cisplatin. D) Kaplan–Meier analysis shows the PFS differences between predicted cisplatin‐sensitive and insensitive patient groups. E) Comparing predicted LN IC50 to cisplatin among patients with different types of cancer. We used a two‐sided Wilcoxon rank‐sum test to assess the differences between each group and all other patients (**** *p* < 1e‐4; ** 1e‐3 < *p* < 1e‐2). F) Kaplan–Meier analysis shows the PFS differences between predicted cisplatin‐sensitive and insensitive CESC patient groups. G,H) Comparing the feature values of dimension 4 (G) and dimension 20 (H) between predicted cisplatin‐sensitive and insensitive patient groups.

In this study, we developed DeepCCDS aimed at predicting the sensitivity of cell lines to drugs. Through previous analyses, we have comprehensively demonstrated the framework's robust predictive performance and interpretability. Here, we further applied it to solid tumor patient samples from TCGA database to explore the model's applicability in real clinical patients. We first obtained patient‐drug combinations with recorded drug responses from the TCGA based on previous research.^[^ ^40^ ^]^ After screening and preprocessing, a total of 1489 patient‐drug combinations were retained, comprising 817 unique patients and 25 unique drugs. We applied DeepCCDS to these combinations to predict their LN IC50 values and compared them with the sensitivity of cell lines to the same 25 drugs in GDSC and found consistency in the prediction results (**Figure** **7**A). Moreover, the predicted LN IC50 of responsive patient‐drug combinations was significantly lower than that of non‐responsive patient‐drug combinations (Wilcoxon's p‐value = 9.1e‐05; Figure 7B).

We next focused on the prediction of patient response to cisplatin, as it is a widely used chemotherapeutic agent playing a crucial role in the treatment of multiple solid tumors.^[^ ^41^ ^]^ We observed DeepCCDS could still accurately predict patient response to cisplatin treatment (Wilcoxon test p‐value = 7.1e‐03; Figure 7C). We then obtained multi‐omics data and clinical information for all TCGA patients who received cisplatin treatment from cBioPortal. After predicting these patients’ sensitivity to cisplatin treatment and dividing them into sensitive and insensitive groups, we found that patients predicted to be sensitive had significantly better progression‐free survival (PFS) compared to insensitive patients (Log‐rank test p‐value = 6.9e‐04; Figure 7D). Previous studies have shown that cervical squamous cell carcinoma (CESC) patients receiving cisplatin treatment exhibit the best progression‐free survival compared to other cancers.^[^ ^42^ ^]^ We compared the predicted LN IC50 of the top five prevalent cancers treated with cisplatin (Figure S17, Supporting Information) and observed that CESC patients indeed had the lowest predicted LN IC50 values (Figure 7E). Survival analysis indicated that CESC patients responsive to cisplatin treatment had markedly better progression‐free survival (Figure 7F).

To further evaluate the clinical application potential of DeepCCDS, we compared it with other state‐of‐the‐art methods designed for drug response prediction. We focused on two representative approaches, DrugFormer^[^ ^43^ ^]^ and SpaRx,^[^ ^44^ ^]^ which are specifically developed to leverage single‐cell data for modeling drug response and have shown strong translational potential. A recent study based on single‐cell analysis provided an in‐depth investigation into cisplatin resistance in bladder cancer patients.^[^ ^45^ ^]^ Therefore, we selected bladder cancer as the disease context and used corresponding single‐cell and spatial transcriptomics data to train DrugFormer and SpaRx (see Methods section). Both models were implemented using the publicly available code provided by the authors on GitHub. Finally, we applied the trained models to predict cisplatin response in TCGA Bladder Urothelial Carcinoma (BLCA) patients and evaluated the predictions against the actual clinical response labels using Fisher's exact test and F1 score. As shown in Figure S18, Supporting Information, DeepCCDS achieved the most significant p‐value and the highest F1 score, demonstrating its superior predictive power and highlighting its promise for clinical applications.

In previous results, we demonstrated that model interpretation could identify feature dimensions characterizing cancer cell sensitivity. Here, we used the IG algorithm to calculate the contribution of feature dimensions to the clinical response to cisplatin. Dim 1 and 15 still showed relatively high contributions, but the most important dimensions were dim 4 and 20 (Figure S19, Supporting Information). We applied dims 4 and 20 to patients receiving cisplatin treatment and found that the feature values of these dimensions significantly differed between the cisplatin‐sensitive and cisplatin‐insensitive groups (Figure 7G,H). These results indicate that, despite the challenges in translating from cell lines to solid tumor patients, DeepCCDS can still provide valuable information for predicting drug responses in solid tumor patients and possess certain clinical relevance and interpretability.

## 3 Discussion

In cancer treatment, predicting individual drug responses is crucial for guiding personalized therapeutic strategies, enhancing treatment efficacy, and reducing unnecessary side effects. However, this task remains challenging due to cancer's complexity. Our study introduces the DeepCCDS framework, which integrates deep learning technology with prior biological network knowledge to predict cancer cell line sensitivity to drugs using multi‐omics features.

We recognize that the mutation status of cancer driver genes plays a crucial role in drug sensitivity prediction, as these mutations often serve as key drivers of cancer initiation and progression. However, cancer is not driven by isolated genetic mutations but rather by the synergistic effects of multiple mutations that perturb cellular signaling networks. Relying solely on individual gene mutations is insufficient to capture the complexity of cancer regulation. To address this, we utilized a prior knowledge‐based network to identify the cascade effects of cancer driver mutations on downstream molecules, referred to as cancer driver signals, and mapped these signals onto biological pathways. Ultimately, we incorporated both driver gene mutations and pathway features to characterize cancer cell lines. While mutations provide the source of oncogenic signals, pathways reveal their functional consequences. This integration effectively captures key regulatory processes and signal transduction cascades, enabling the model to develop a more precise understanding of cancer regulatory mechanisms.

In a comprehensive evaluation using cell‐drug paired data from the GDSC database, DeepCCDS demonstrated remarkable accuracy in predicting drug sensitivity, achieving a high PCC and low RMSE. The model's robustness was validated through multiple random data splits, while its generalizability was confirmed on independent external datasets CCLE and NCI60. Through comparison, DeepCCDS outperformed other deep learning methods and traditional machine learning algorithms in overall prediction accuracy and in predicting sensitivity for specific cells or drugs (Figure 3 and Figures S5–S8, Supporting Information). Furthermore, our approach achieves high predictive performance while maintaining faster training speed and lower memory consumption (Figure S12, Supporting Information). This can be attributed to two main factors: first, the use of relatively lightweight model architecture, and second, our approach's ability to distill high‐dimensional information from a large and complex prior knowledge network into biologically meaningful, low‐dimensional representations. This process significantly reduces the computational and memory demands during the subsequent model training phases.

Through deep learning techniques, DeepCCDS successfully generated low‐dimensional embedded features of cells, which outperformed original high‐dimensional features in predicting drug sensitivity. Visualization using t‐SNE algorithm confirmed these embedded features' ability to distinctly separate sensitive and insensitive cell populations, indicating high‐quality feature representation. Comprehensive model interpretation based on these high‐quality features revealed complex biological mechanisms underlying drug responses. Specifically, we observed that some pathways, such as the Insulin secretion pathway, ErbB signaling pathway, Estrogen signaling pathway, and Natural killer cell‐mediated cytotoxicity, with high contributions to predictions indeed participate in the regulation of drug sensitivity.^[^ ^27^, ^28^, ^29^, ^30^ ^]^ The activity of these pathways shows significant differences between sensitive and insensitive cell populations. For mutation embedding features, represented by 30 neurons in the bottleneck layer, we performed pathway annotation to link abstract mathematical representations with actual biological processes. The unique annotations for each dimension reflected DeepCCDS's capability to capture key variations in the original data. Notably, we found that dimensions 1 and 15 are highly correlated with cell sensitivity to drugs, and they exhibit completely opposite trends. Leveraging the feature values, these two dimensions have shown potential in inferring drug indications.

We applied DeepCCDS to patient‐drug combinations in the TCGA database, successfully extending the model's predictive capabilities from cell lines to clinical patients. The predicted drug sensitivity demonstrated significant concordance with the actual clinical response. This capability is crucial for guiding personalized treatment decisions. We used cisplatin as a case study to illustrate that DeepCCDS can predict the clinical responses of patients to specific treatments. To further validate the accuracy of the predictions, we conducted survival analyses. The results indicated that patients predicted to be sensitive exhibited significantly better PFS, strongly supporting DeepCCDS's prediction in clinical patients. In specific cancer types, the model's predictions were highly consistent with known clinical observations, underscoring DeepCCDS's ability to capture cancer‐specific characteristics, which is essential for optimizing treatment strategies. Through model interpretation, the study identified feature dimensions that significantly contribute to clinical responses to cisplatin. This provides new insights into the mechanisms of drug response and may serve as new biomarkers for patient stratification and treatment response prediction.

However, the dependency of DeepCCDS on the quality of prior knowledge networks still exists. Although we used a high‐quality network in this study, we fully acknowledge that prior knowledge networks are continually evolving and may not capture all relevant or context‐specific regulatory events. In future work, we plan to integrate more comprehensive and up‐to‐date interaction databases to improve network completeness. Additionally, we will explore confidence‐weighted to better handle uncertainty and variability in the quality of prior knowledge, thereby enhancing the reliability of our model. In conclusion, DeepCCDS successfully combines deep learning techniques with prior biological knowledge, offering a novel perspective for understanding and predicting cancer treatment responses. DeepCCDS goes beyond the conventional use of isolated genetic markers by characterizing cancer driver signals as biological entity representations using prior biological knowledge. Comprehensive validation and evaluation have shown that DeepCCDS outperforms existing state‐of‐the‐art methods in predicting drug sensitivity in cancer cell lines, with significant potential in drug repurposing and clinical decision‐making. We believe this study can bridge the gap between complex biological systems and computational techniques, aiming to revolutionize drug sensitivity prediction and pave the way for truly personalized cancer treatment.

## 4 Experimental Section

### Preparing Data from GDSC

This work utilized the extensive cancer cell line and drug resources provided by the GDSC database (https://www.cancerrxgene.org) as our training data. From the mutation annotation files of cell lines in the database, this work retained only the mutation information of experimentally validated cancer driver genes collected from the COSMIC Cancer Gene Census (CGC).^[^ ^46^ ^]^ The annotation files in GDSC were converted into a binary mutation matrix, with rows and columns representing genes and cells, respectively, using 1 and 0 to indicate non‐synonymous mutations and wild type. This work then obtained the TPM (transcript per million) normalized gene expression matrix and applied a log transformation. Only cell lines common to both matrices were used, and this work removed cancer types with fewer than 10 cell lines, resulting in 866 cell lines covering 22 cancer types. This work had provided the detailed category distribution of these cell lines in the Supplementary Materials (Figure S20A, Supporting Information).

For drug features, this work queried Simplified Molecular Input Line Entry System (SMILES) strings for drugs using the Python library PubChemPy. Some drugs with no matches were manually annotated. Finally, SMILES strings were successfully retrieved for 413 drugs. These SMILES strings were then converted into Morgan molecular fingerprints of size 1024 using the R package “rcdk” (https://cran.r‐project.org/web/packages/rcdk/), serving as molecular structure features for the drugs. Like the cell lines, these drugs also encompass various types, including different anatomical classifications (ATC) and mechanisms of action. This work annotated the functional information of these drugs using the ChEMBL database (Table S4, Supporting Information).

This work paired all cell lines and drugs, matching drug sensitivity information from GDSC1 and GDSC2. The natural logarithm of the half‐maximal inhibitory concentration (LN IC50) was chosen as the measure of drug sensitivity. For cell‐drug pairs duplicated in GDSC1 and GDSC2, the LN IC50 value from the newer version (GDSC2) was retained. After all preprocessing steps, a total of 319 543 cell‐drug pairs were retained. This work used 80% of all cell‐drug pairs (255 628 pairs) as a training set, 10% as a validation set (31 963 pairs) to monitor the training process to prevent overfitting, and 10% (31 952 pairs) as a test set to evaluate performance (Figure 1A).

### Preparing External Validation Sets

This work selected material resources provided by the CCLE database (https://sites.broadinstitute.org/ccle)^[^ ^7^ ^]^ and NCI60 dataset^[^ ^47^ ^]^ for external validation of our model. The NCI60 dataset was obtained from the R packages rcellminer (https://bioconductor.org/packages/release/bioc/html/rcellminer.html) and rcellminerData (https://bioconductor.org/packages/release/data/experiment/html/rcellminerData.html). For the drugs in the NCI60 dataset, this work directly utilized the SMILES information provided in the rcellminerData package. Both CCLE and NCI60 datasets underwent the same preprocessing as the GDSC data for all cell and drug features. Finally, the CCLE dataset retained 10 778 cell‐drug pairs, encompassing 466 cell lines and 24 drugs. NCI60 contains a diverse array of over 50 000 drugs. This work retained only 867 drugs that were FDA‐approved and had accessible SMILES information. The NCI60 dataset preserved 50 055 cell‐drug pairs, covering 60 cell lines and 867 drugs (Figure 1A). This work also presented detailed information on the cell lines and drugs in the Supplementary Materials (Figure S20B,C and Tables S5 and S6, Supporting Information).

### Preparing Datasets for Evaluating Clinical Potential

This work obtained TCGA patient‐drug combinations with documented drug response records from a previous study by Ding et al.^[^ ^40^ ^]^ This study had already standardized the initially inconsistent drug name records. Clinical responses were categorized according to the Response Evaluation Criteria in Solid Tumors (RECIST) standard^[^ ^48^ ^]^ as responders (including complete response and partial response) and non‐responders (including stable disease and disease progression). After filtering for patients with both expression and mutation data available, this work retained a final dataset comprising 1489 patient‐drug combinations, involving 817 unique patients and 25 distinct drugs. This work then collected data on 557 cisplatin‐treated TCGA patients via cBioPortal.^[^ ^49^ ^]^ These patients had multi‐omics features (including gene expression and mutation information) and clinical information but lacked known drug response labels. This work further obtained scRNA‐seq data from bladder cancer patients treated with cisplatin (GSE192575^[^ ^45^ ^]^) for model training of the DrugFormer^[^ ^43^ ^]^ method. This work extracted gene expression profiles of bladder cancer cell lines treated with cisplatin from the GDSC database and utilized a dataset (VISDP000028) from the CROST database^[^ ^50^ ^]^ that includes both single‐cell RNA sequencing and spatial transcriptomics data from bladder cancer patients for model training of the SpaRx^[^ ^44^ ^]^ method.

### Design of DeepCCDS—The framework Overview

DeepCCDS is a novel deep learning framework designed to predict drug sensitivity for certain cell‐drug pairs by integrating cellular features (gene expression and mutation profiles) and drug features (molecular fingerprints). The framework comprised four main components: a prior knowledge network, two autoencoders, and a feedforward neural network. The prior knowledge network was employed to characterize cancer driver signals as pathways (Figure 1B). Pathway activities were utilized as learned embedded representations of the cell's gene expression profile. The mutation and drug autoencoders were used to learn embedded representations of driver gene mutation states and molecular fingerprints, respectively. These three embedded vectors were then concatenated and fed into the feedforward neural network to generate a predicted drug sensitivity value, expressed as the LN IC50 (Figure 1C). The training process of DeepCCDS is divided into two main stages: 1) the pre‐training stage, which involves characterizing cancer driver signals as pathway representations and determining parameters of neural networks; 2) the complete training stage, in which the entire DeepCCDS framework undergoes end‐to‐end training using features and the determined parameters.

### Characterizing Cancer Driver Signals through a Prior Knowledge Network

A crucial component of DeepCCDS was characterizing cancer driver signals as biological pathways (Figure 1B). This work first captured the cancer driver signals via the prior knowledge network. To do this, this work employed a comprehensive human protein‐protein interaction (PPI) network curated by a previous study,^[^ ^51^, ^52^ ^]^ which integrates data from 12 different sources. This integration helps ensure broad coverage of biologically meaningful interactions. To further enhance the reliability of the network, this work retained only those interactions supported by at least two independent sources, thus ensuring high‐confidence associations. The largest connected subgraph, extracted using the R package “igraph,” comprised 12 436 gene nodes and 83 020 edges. This work defined an adjacency matrix *P* to represent this PPI network, where rows and columns correspond to gene symbols, and binary values 1 and 0 indicate the presence or absence of edges between genes, respectively. Diagonal elements were set to 0 to eliminate self‐connections.

Cancer driver genes obtained from the CGC were mapped onto the PPI network. A total of 534 genes were successfully mapped and used as seed nodes to compute their extensive influence on other genes in the network using the Random Walk with Restart (RWR) algorithm. This work defined a probability vector *c^0^* to represent the initial state of genes in the network before RWR. All driver genes were assigned equal probabilities summing to 1, while all other genes were initialized with a probability of 0. The RWR process is represented by the adjacency matrix *P*, the initial probability vector *c*, and an iterative diffusion function:

(1) $$c^{t+1}=\left(1-\beta \right)\;Tc^{t}+\beta c^{0},\mathrm{where}\;\;T_{ij}=\frac{P_{ij}}{\sum_{j=1}^{N_{g}}P_{ij}}$$

Here, *T* is the probability transition matrix obtained by column‐normalizing matrix *P*. *T_ij_* represents the probability of signal transmission from gene *j* to gene *i*. *Ng* denotes the total number of genes in the network. *β* is the restart probability, controlling diffusion depth, set to 0.9 in this study. *c^t^* corresponds to the probability vector encompassing node probabilities at step *t*. The iteration stops when the difference between *c^t+1^* and *c^t^* is less than 1e‐10. The final probability *c^i^* in vector *c* represents the strength of the signal transmitted to gene *i*. For characterizing the signals as pathway representations, we obtained 323 pathways from the KEGG database.^[^ ^53^ ^]^ Using these pathways and the gene ranked list *c*, we applied Gene Set Enrichment Analysis (GSEA) to calculate enrichment scores (ES) and statistical significance for all pathways. Based on criteria of ES > 0 and FDR < 0.2, we ultimately characterize driver signals with 38 pathways. Upon inputting the gene expression matrix of cell lines into DeepCCDS, it was first transformed into an activity score matrix for these 38 pathways using the single sample GSEA (ssGSEA) algorithm. The pathway activity score can reflect the cellular environment after perturbation by driver genes, which is then used for drug sensitivity prediction (Figure 1C).

### Parameter Setting of Mutation and Drug Fingerprint Autoencoders

DeepCCDS employs autoencoders to learn embedded representations of cell mutation information and drug molecular fingerprints. This work first pre‐trained two autoencoders to determine the optimal parameters for these encoding networks. The input dimensions were 534 for the mutation autoencoder and 1024 for the drug autoencoder. Each autoencoder consisted of a three‐layer encoder (two hidden layers and one bottleneck layer) and a symmetric decoder. Each layer incorporated batch normalization to enhance model generalization and uses the Rectified Linear Unit (ReLU) activation function to handle non‐linearity. Each decoder's output layer incorporated a sigmoid activation function to ensure that the outputs range between 0 and 1. This work defined the parameter space for the number of neurons in each layer as follows: hidden layer 1 {300, 200, 100}, hidden layer 2 {100, 50, 30}, bottleneck layer {30, 20, 10}. A grid search method was employed to explore different parameter combinations within this fixed parameter space. For each autoencoder, we trained every parameter combination for 50 epochs using the training set. The model was implemented and optimized by ADAM optimizer in the Python library PyTorch 2.1.0. This work used the binary cross‐entropy (BCE) as the loss function to optimize the autoencoder. The parameter combination yielding the lowest reconstruction BCE on the test set was selected for the complete DeepCCDS training (300, 100, and 30 neural, respectively; Figure S1, Supporting Information).

### Parameter Setting of Feedforward Neural Network

The feedforward neural network was designed to predict drug sensitivity for specific cells. It comprised an input layer consisting of a concatenated vector of embedded representations, two hidden layers each with the same number of neurons as the input layer, and an output layer with a single neuron without an activation function. The input layer integrated the embedded representations of gene expression, mutations, and molecular fingerprints. Each hidden layer included batch normalization and uses the ReLU activation function. The network's output was designed to fit the LN IC50 value, representing the cell's sensitivity to the drug. The absence of an activation function in the output layer allowed for unrestricted prediction of the continuous LN IC50 value.

### Complete Training and Evaluation

The complete DeepCCDS framework inputs gene expression and mutation profiles of cell lines and molecular fingerprints of drugs and predicts drug sensitivity (LN IC50) as output. This work randomly selected 80% of all GDSC cell‐drug pairs (255 628 pairs) as a training set, 10% as a validation set (31 963 pairs) to monitor the training process to prevent overfitting, and 10% (31 952 pairs) as a test set to evaluate performance. The training process was implemented in an end‐to‐end manner using PyTorch 2.1.0 and optimized by ADAM optimizer. The optimization objectives of the model encompass two primary components: minimizing the loss between predicted and observed drug sensitivities and minimizing the reconstruction loss of the autoencoders. The inclusion of reconstruction losses encouraged the network to extract meaningful encodings from the input cell line and drug features. A unified loss function encapsulated all the optimization objectives, which is iteratively minimized across training batches to calibrate the model parameters:

(2)

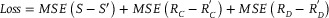

where *S* and *S*′ denote the observed and predicted drug sensitivities, respectively. *R_C_* and $R_{C}^{\prime }$ represent the input and reconstructed features of the mutation autoencoder. Similarly, *R_D_* and $R_{D}^{\prime }$ signify the input and reconstructed features of the drug autoencoder. This work then employed a grid search on the training set to determine the optimal hyperparameters. The hyperparameter space included: learning rates {1e‐2, 1e‐3, 1e‐4}, batch sizes: {256, 512, 1024}. This work trained every parameter combination for 50 epochs. The combination with the lowest PCC and RMSE between predicted and observed LN IC50 values in the test set was selected (learning rate = 1e‐3, batch size = 1024; Figure S2, Supporting Information). This work then trained the model using the selected hyperparameters. To mitigate overfitting, this work implemented early stopping with a patience of 10 epochs (i.e., training stopped if the validation loss did not improve for 10 consecutive epochs). The maximum number of epochs was set to 100. This work assessed DeepCCDS's predictive performance on both the test set and external validation datasets (CCLE and NCI60) with PCC and RMSE between predicted and observed LN IC50 values. To assess the model's robustness, we conducted Monte Carlo cross‐validation with 10 iterations, each using a random 8:1:1 split of data for training, validation, and testing.

### Benchmarking Drug Sensitivity Prediction

To evaluate the performance of DeepCCDS in the context of existing approaches, this work conducted a comprehensive benchmarking study. This work compared DeepCCDS against well‐cited deep learning frameworks, including DeepTTA,^[^ ^9^ ^]^ DeepDR,^[^ ^10^ ^]^ Precily,^[^ ^1^ ^]^ BANDRP,^[^ ^22^ ^]^ DeepCDR,^[^ ^23^ ^]^ and DrugCell,^[^ ^24^ ^]^ as well as several traditional machine learning algorithms. All models were trained and tested on the same datasets used for DeepCCDS to ensure a fair comparison. This work implemented each deep learning framework using the code provided in their respective publications. For a more thorough assessment and benchmarking of the model's predictive capability, this work used evaluation metrics based on regression (PCC and RMSE) and classification (AUROC and F1 score). For the calculation of classification metrics, this work classified cell‐drug pairs into sensitive and insensitive groups using the top quartile threshold of the actual IC50 values. This work then applied this threshold to the predicted IC50 values to assign each sample to a sensitive or insensitive group. Based on this classification, this work computed the AUROC and F1 scores to evaluate prediction performance. DeepDR was designed to predict sensitivity for a fixed set of 265 drugs due to its architectural constraints. For a fair comparison, this work used the fully trained DeepCCDS to generate predictions for only these 265 drugs, labeling this subset of predictions as “DeepCCDS265.” This work also benchmarked against traditional machine learning algorithms, including lasso, ridge, elastic net regression models, and support vector machines (SVM). These were implemented using the “glmnet” package in the R environment.

### Model Interpretation—Feature Importance Evaluation

DeepCCDS takes two types of cell line features as input: gene expression and mutation profiles. The gene expression profiles were embedded into 38 pathways, while the gene mutation profiles were embedded into 30 bottleneck layer neurons. To explore the relationship between each embedded feature and drug sensitivity, this work employed the Integrated Gradients (IG) method.^[^ ^26^ ^]^ IG attributes the model's prediction for its input features by computing gradients for each input and measures the change in the output based on the small changes in the input. This work calculated the average attribution of features across all samples to represent the global importance, termed the IG score. The calculation was performed through the “IntegratedGradients” class from the Python “Captum” library. Negative IG scores suggested that the feature reduces the IC50 value, indicating increased drug sensitivity because lower IC50 values mean higher drug efficacy. Conversely, positive IG scored imply the feature decreased drug sensitivity.

### Biological Annotation of Mutation Embedding Features

To interpret the biological significance of the 30D embedded features derived from driver gene mutations, this work developed an annotation process. Let *E* be the matrix of mutation embedding features and *G* be the gene expression matrix. The rows of matrix *E* represent 30 embedded features, while the rows of matrix *G* represent genes. This work calculated the correlation between each row of matrices 𝐸 and *G*, resulting in matrix *C*. Here, *C_ij_* is the PCC between the *i^th^* feature dimension and the expression of *j^th^* gene. For each row of matrix *C*, this work performed a descending sort and conducted GSEA using 323 KEGG pathways. Based on FDR < 0.01, this work filtered the biological pathway annotations related to each feature dimension.

## Conflict of Interest

The authors declare no conflict of interest.

## Author Contributions

J.W., J.L., X.Z., should be regarded as joint first authors. Conceptualization, J.H. and J.W.; methodology, J.L. and J.W.; validation, J.L. and Y.S.; formal analysis, Y.S. and X.Z.; investigation, J.W. and J.H.; resources, Y.H. and L.W.; data curation, S.L. and Z.W.; writing—original draft preparation, J.L. and J.W.; writing—review and editing, J.H.; visualization, J.W. and Y.Z.; supervision, J.H. and Y.J.; project administration, J.H.; funding acquisition, J.H. All authors have read and agreed to the published version of the manuscript.

## Supporting information



Supporting Information

Supporting Information

Supporting Information

Supporting Information

Supporting Information

## Data Availability

All data used in this study are publicly available. Drug response and multi‐omics data of cell lines were obtained from GDSC (https://www.cancerrxgene.org)^[^ ^6^ ^]^ CCLE (https://sites.broadinstitute.org/ccle)^[^ ^7^ ^]^ and the NCI‐60^[^ ^47^ ^]^ panel. The drug structure information was obtained from the PubChem database (https://pubchem.ncbi.nlm.nih.gov/)^[^ ^54^ ^]^ and the R “rcellminerData” package. The TCGA patient data were downloaded from the cBioPortal database (https://www.cbioportal.org).^[^ ^49^ ^]^ TCGA patient‐drug combinations with documented drug response records were obtained from a previous study by Ding et al.^[^ ^40^ ^]^ Pathway data and cancer driver genes were obtained from the KEGG (https://www.genome.jp/kegg)^[^ ^53^ ^]^ and COSMIC CGC (https://cancer.sanger.ac.uk/cosmic)^[^ ^46^ ^]^ databases, respectively, while the PPI network was sourced from previous studies.^[^ ^51^, ^52^ ^]^ The source code of DeepCCDS is available at https://github.com/hanjunwei‐lab/DeepCCDS.
