# Supplementary material for: DeepCCDS: Interpretable Deep Learning Framework for Predicting Cancer Cell Drug Sensitivity through Characterizing Cancer Driver Signals
Source: Adv Sci (Weinh). 2025 May 21;12(23):2416958. doi: 10.1002/advs.202416958 (PMC12199323; doi:10.1002/advs.202416958)
Supplement: Supplementary file 1 — Supporting Information [file ADVS-12-2416958-s003.pdf]

ADVANCED SCIENCE
Open Access

Supporting Information

for *Adv. Sci.*, DOI 10.1002/advs.202416958

DeepCCDS: Interpretable Deep Learning Framework for Predicting Cancer Cell Drug Sensitivity through Characterizing Cancer Driver Signals

*Jiashuo Wu, Jiyin Lai, Xilong Zhao, Ziyi Wang, Yongbao Zhang, Liqiang Wang, Yinchun Su, Yalan He, Siyuan Li, Ying Jiang and Junwei Han**

Supplementary Figures for "DeepCCDS: Interpretable deep learning framework for predicting cancer cell drug sensitivity through characterizing cancer driver signals"

This PDF file includes:

Supplementary Figure S1-20

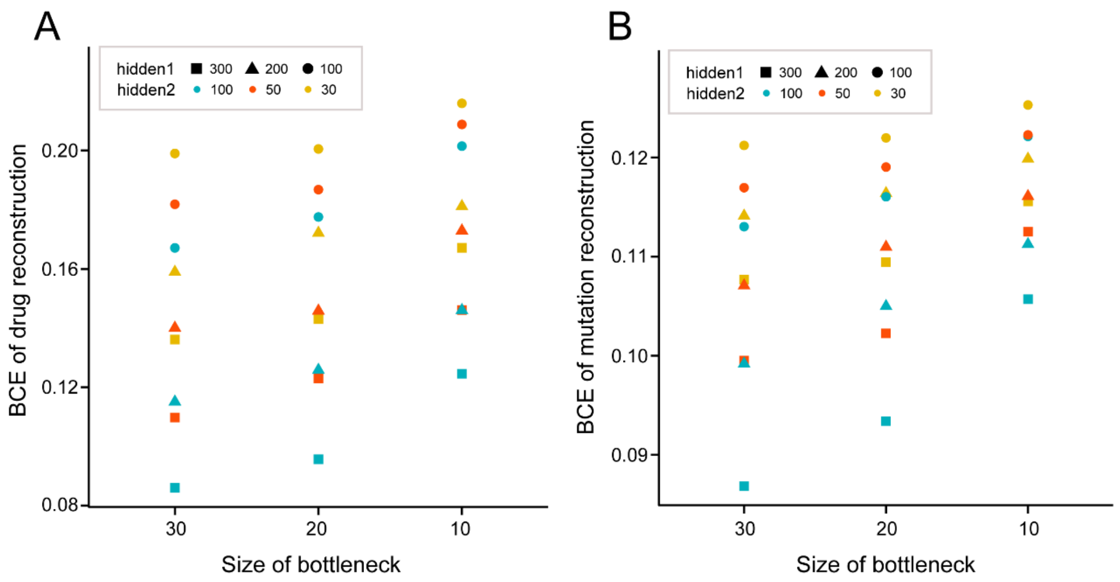

Supplementary Figure S1. (A-B) The scatter plot shows the Binary cross-entropy (BCE) between the input vectors and reconstruction vectors of (A) drug autoencoder and (B) mutation autoencoder under different parameter combinations. The shape of the scatter points represents the dimensions of the first hidden layer, while the color indicates the dimensions of the second hidden layer. The x-axis represents the dimensions of the bottleneck layer.

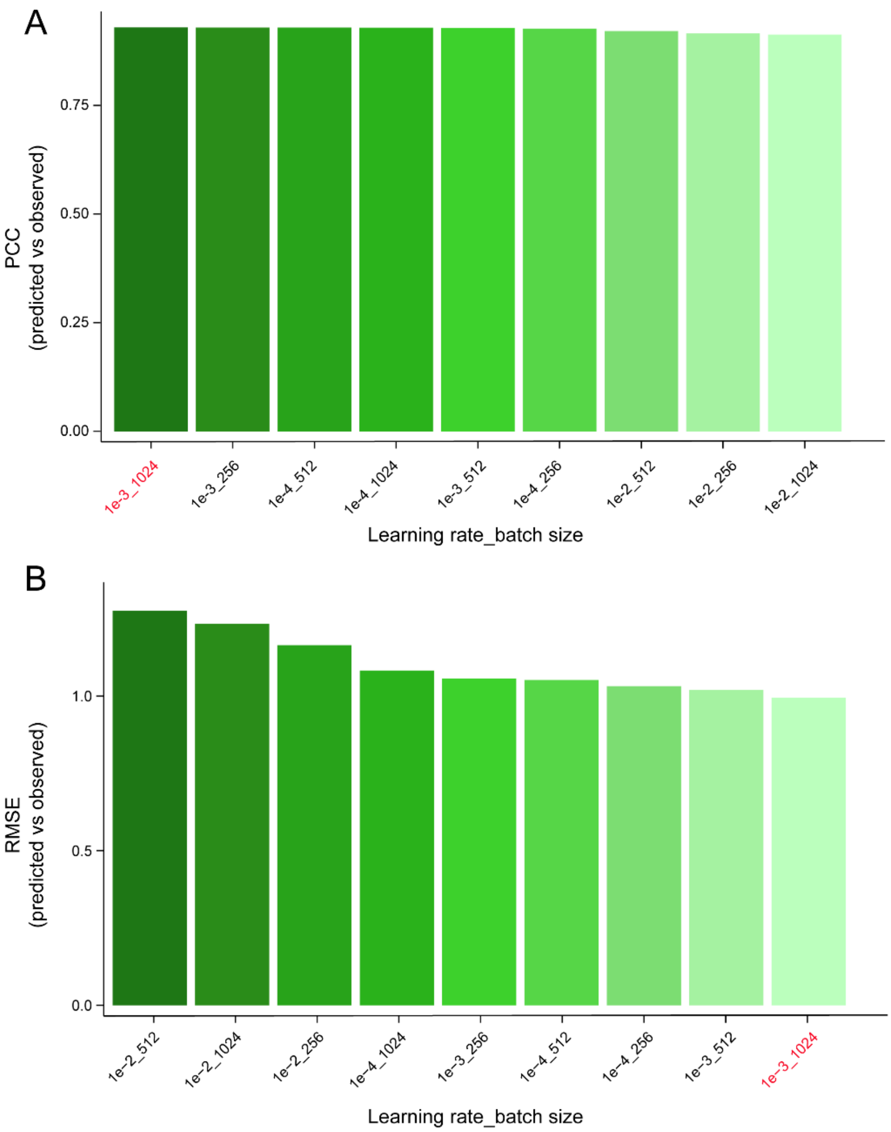

Supplementary Figure S2. (A-B) The bar chart shows the (A) Pearson correlation coefficient (PCC) and (B) the root mean square error (RMSE) between predicted and observed drug sensitivity under different parameter combinations. The parameter space included: learning rates {1e-2, 1e-3, 1e-4}, batch sizes: {256, 512, 1024}. We ultimately selected the parameter combination with the highest PCC and the lowest RMSE (highlighted in red).

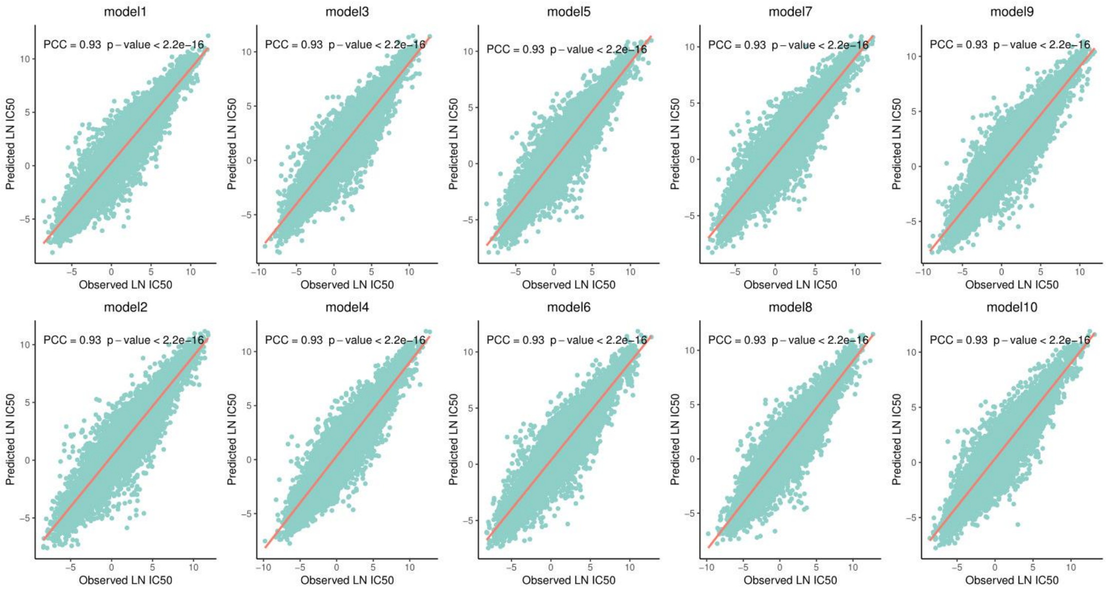

Supplementary Figure S3. The Correlation scatter plot demonstrates the predictive performance of ten models obtained through Monte Carlo cross-validation.

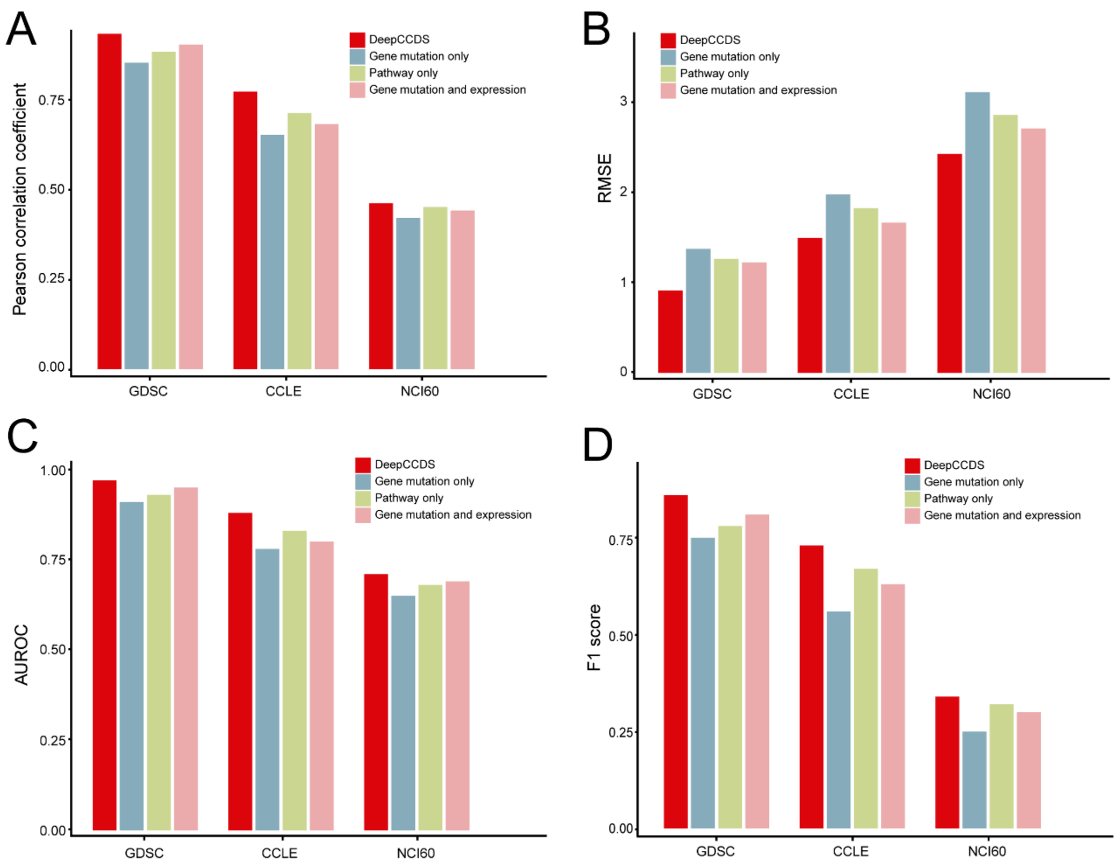

Supplementary Figure S4. Performance comparison of DeepCCDS and its ablated variants across GDSC, CCLE, and NCI-60 datasets using different feature combinations. Models were trained using driver mutations and regulated pathways (DeepCCDS), mutation only, pathway only, and mutation and gene expression data. Performance was evaluated using **(A)** Pearson correlation, **(B)** RMSE, **(C)** AUROC, and **(D)** F1 score.

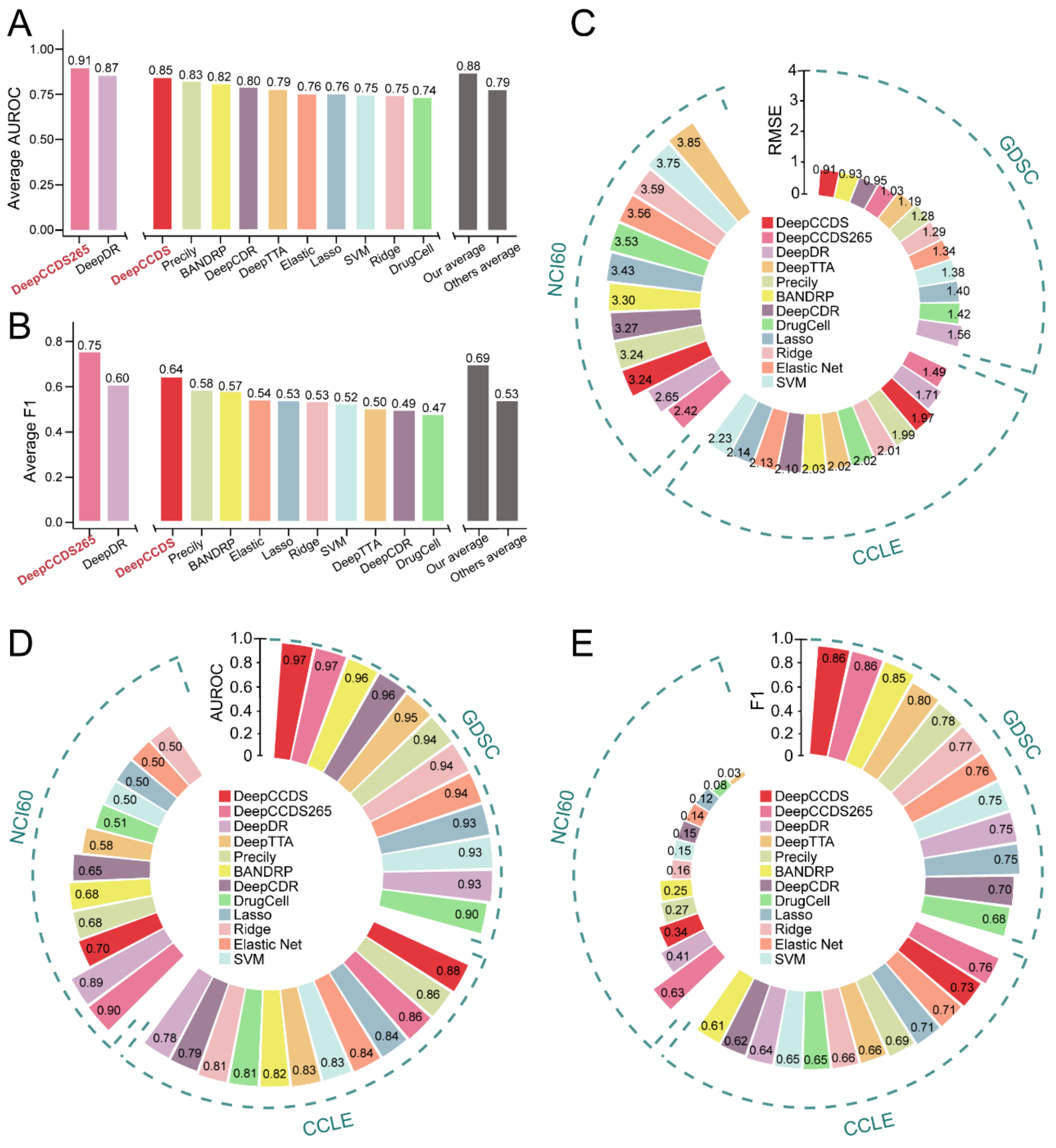

Supplementary Figure S5. (A-B) Comparison of overall performance (average AUROC or F1 score) in different approaches across three datasets. "Our average" refers to the mean overall performance of DeepCCDS and DeepCCDS265, while "Other average" refers to the mean overall performance of the other methods. **(C-E)** The detailed RMSE, AUROC and F1 score of different approaches in three datasets.

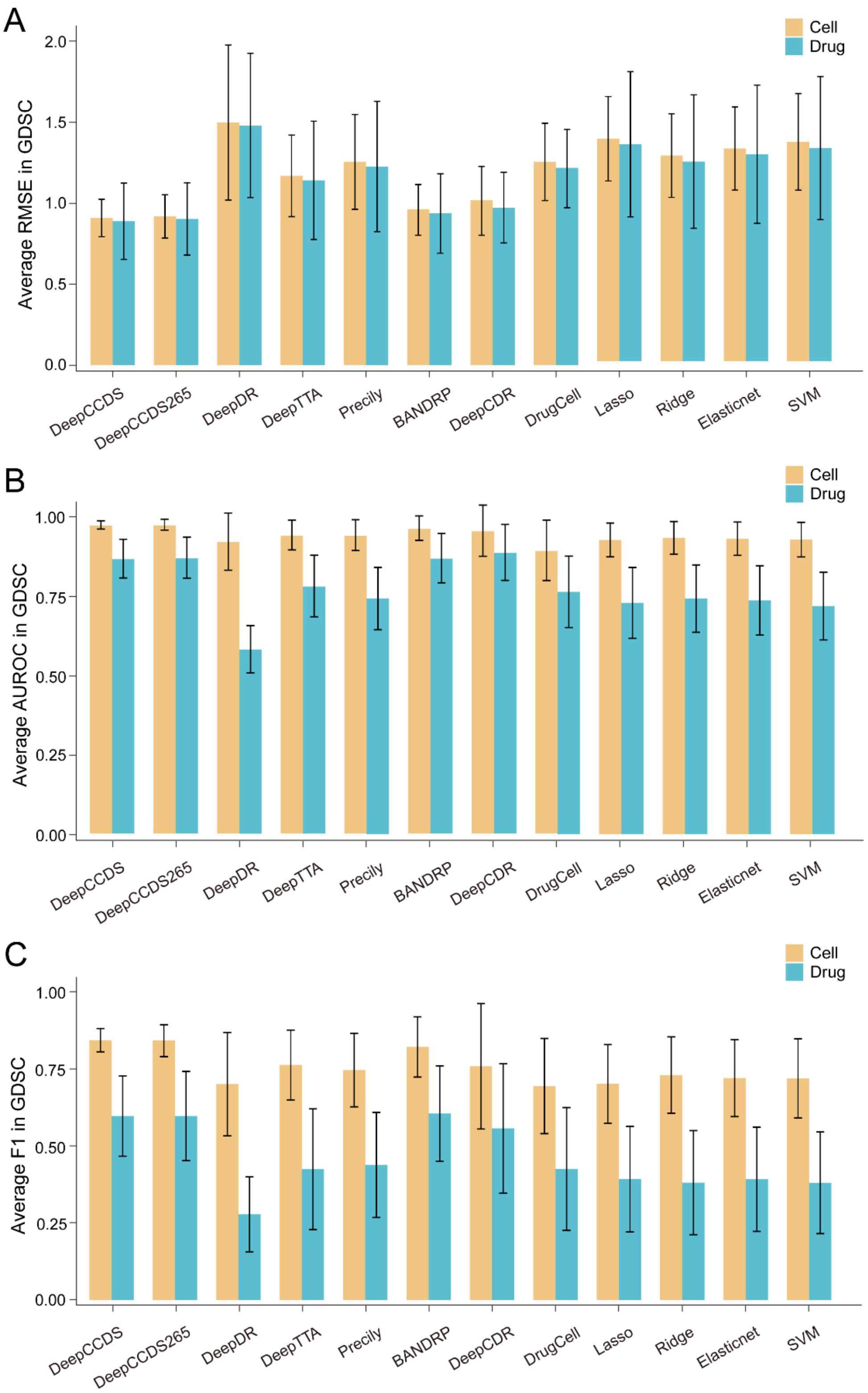

Supplementary Figure S6. Bar charts show the comparison of **(A)** RMSE, **(B)** AUROC and **(C)** F1score of different methods in the GDSC dataset, showing the mean (bars) and standard deviation (error bars) of prediction performance for each cell line across all drugs and for each drug across all cell lines.

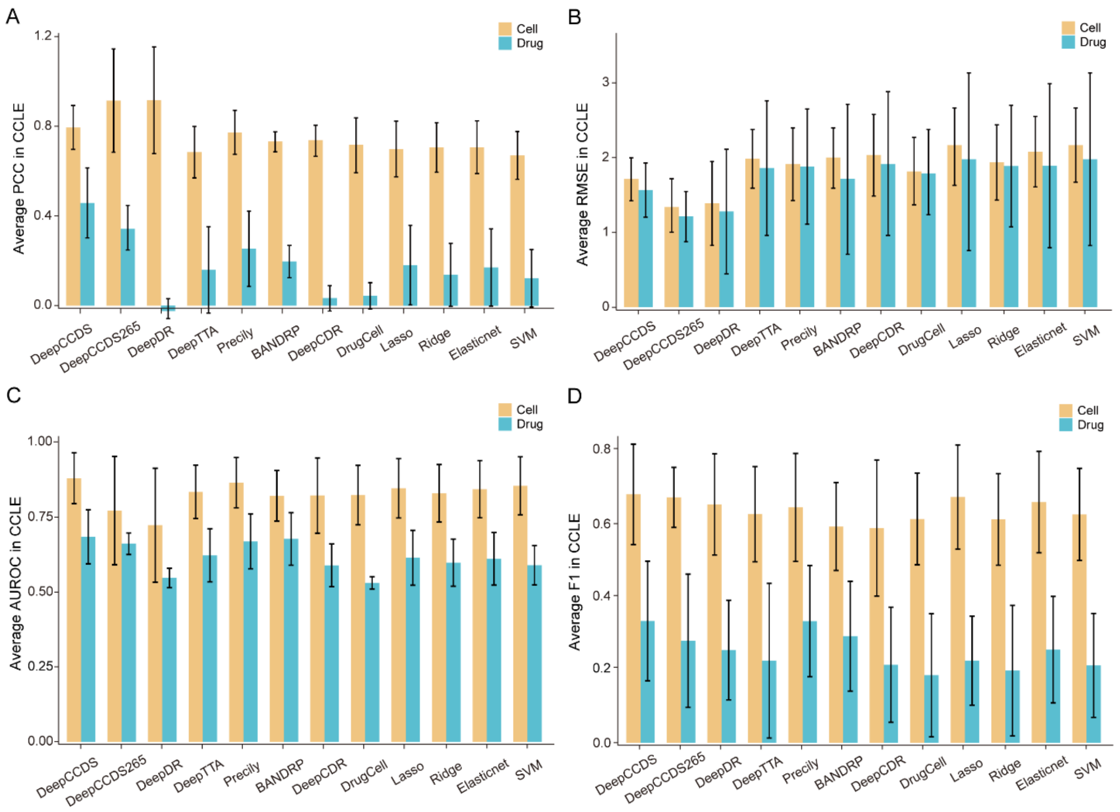

Supplementary Figure S7. Bar charts show the comparison of **(A)** PCC, **(B)** RMSE, **(C)** AUROC and **(D)** F1 score of different methods in the CCLE dataset, showing the mean (bars) and standard deviation (error bars) of prediction performance for each cell line across all drugs and for each drug across all cell lines.

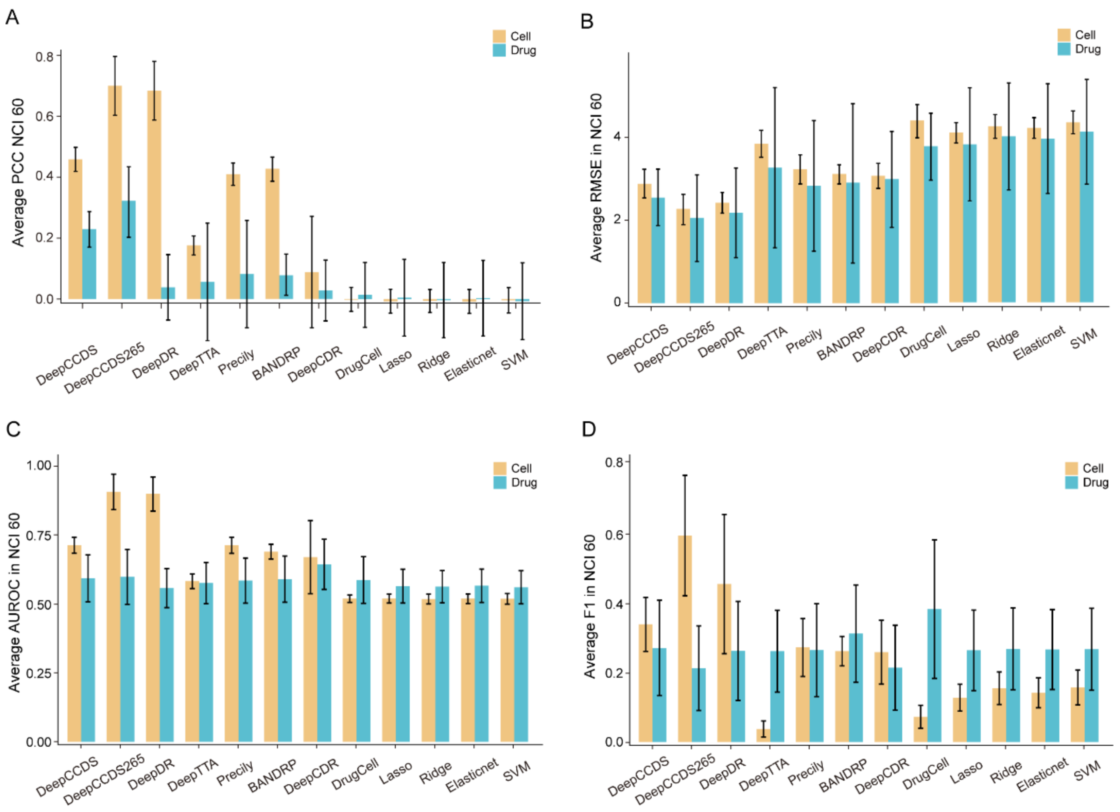

Supplementary Figure S8. Bar charts show the comparison of **(A)** PCC, **(B)** RMSE, **(C)** AUROC and **(D)** F1 score of different methods in the NCI 60 dataset, showing the mean (bars) and standard deviation (error bars) of prediction performance for each cell line across all drugs and for each drug across all cell lines.

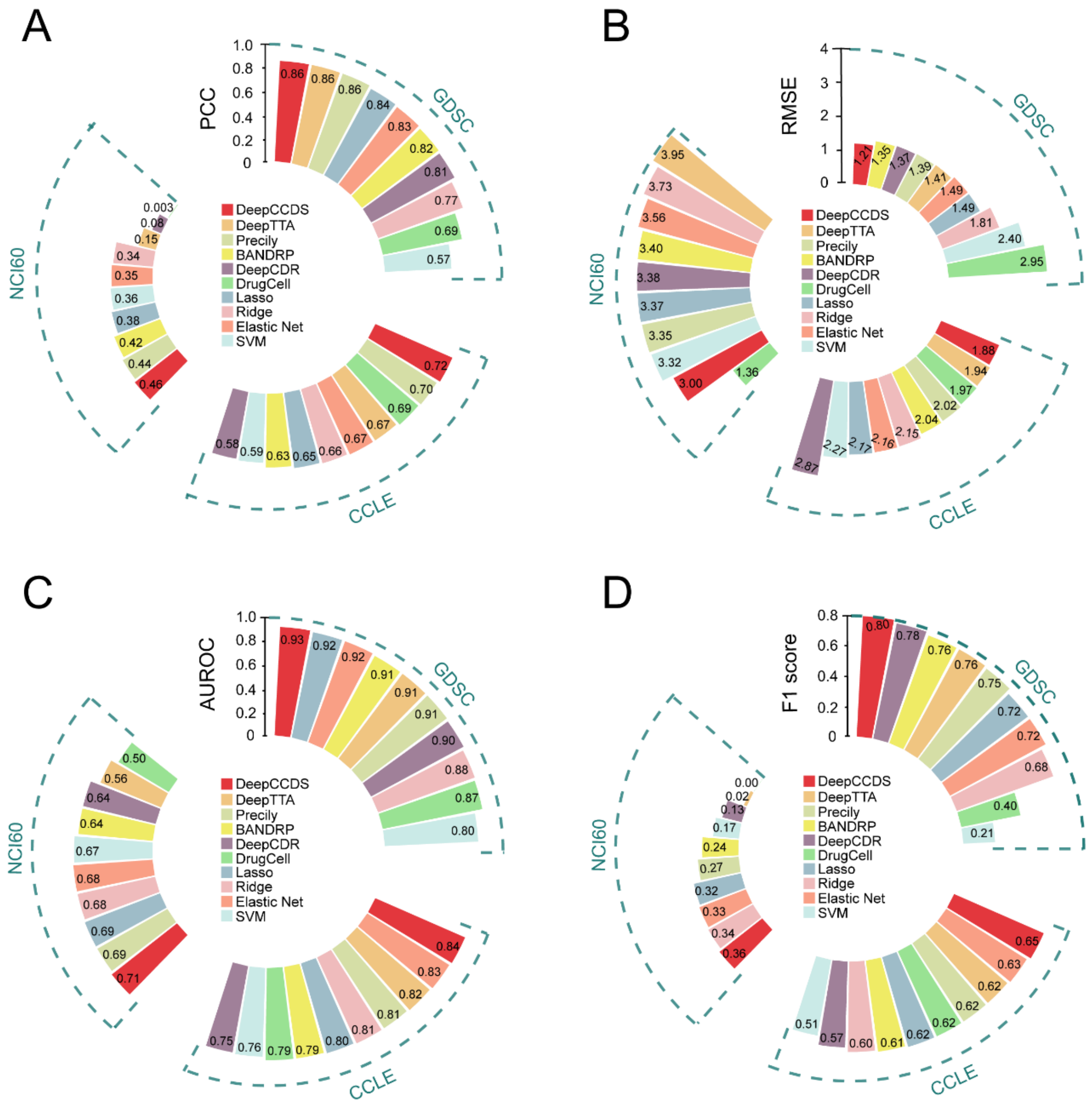

Supplementary Figure S9. The detailed **(A)** PCC, **(B)** RMSE, **(C)** AUROC and **(D)** F1 score of different approaches across three datasets under the "cell split" strategy.

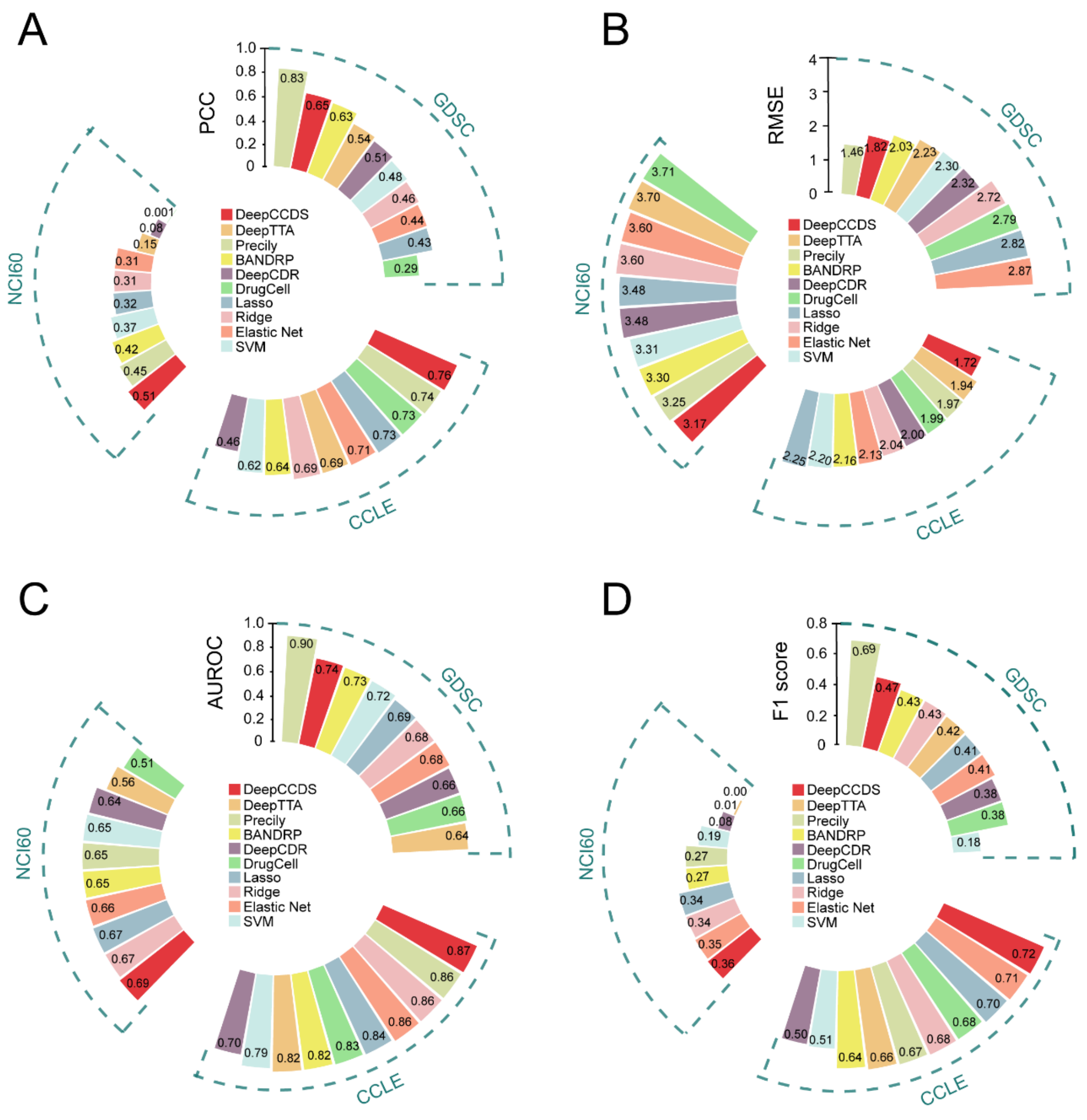

Supplementary Figure S10. The detailed **(A)** PCC, **(B)** RMSE, **(C)** AUROC and **(D)** F1 score of different approaches across three datasets under the "drug split" strategy.

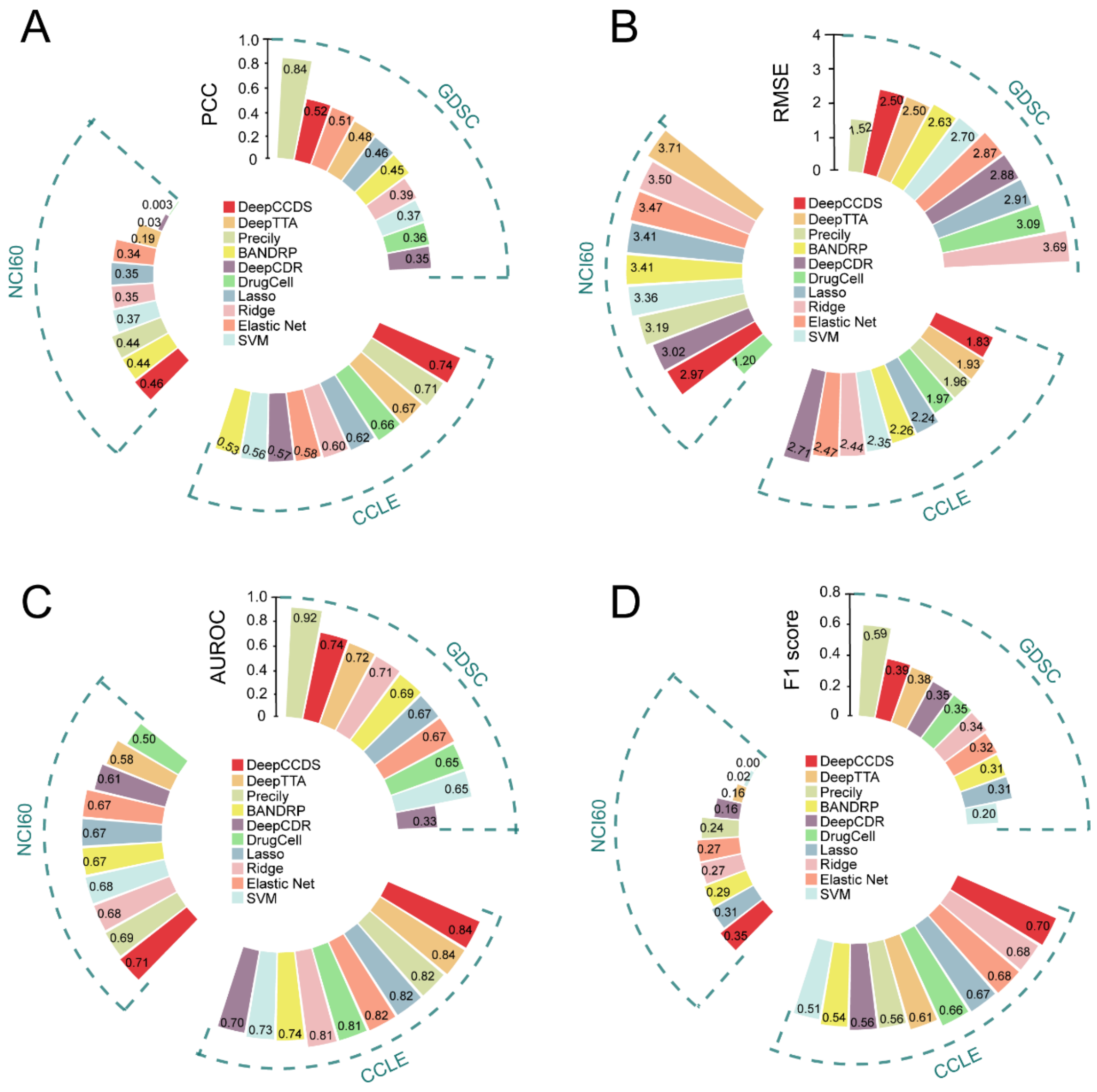

Supplementary Figure S11. The detailed **(A)** PCC, **(B)** RMSE, **(C)** AUROC and **(D)** F1 score of different approaches across three datasets under the "both split" strategy.

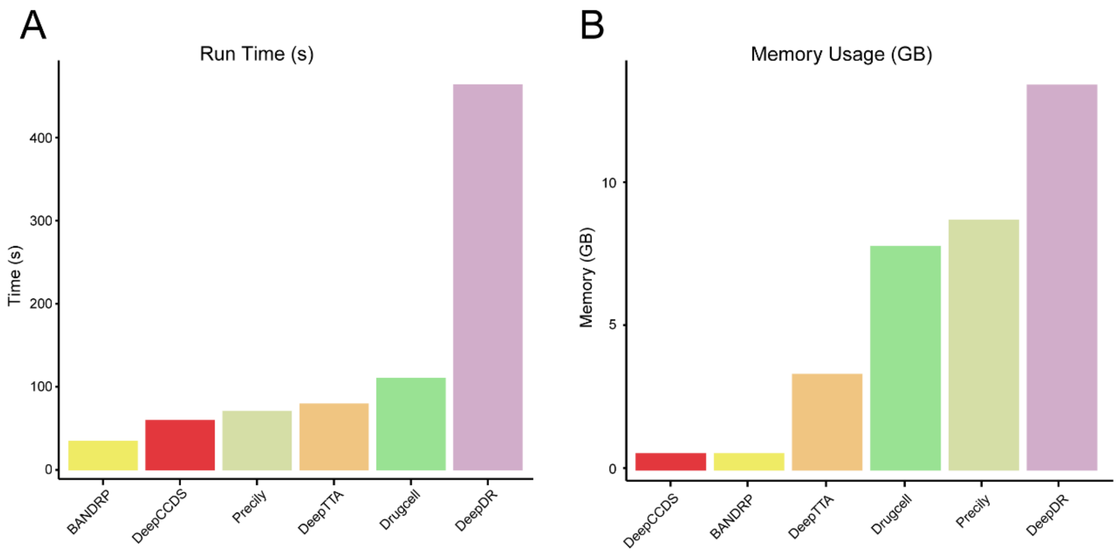

Supplementary Figure S12. The bar chart presents a comparison of the computational efficiency of different deep learning methods, including **(A)** the runtime of each epoch and **(B)** memory usage.

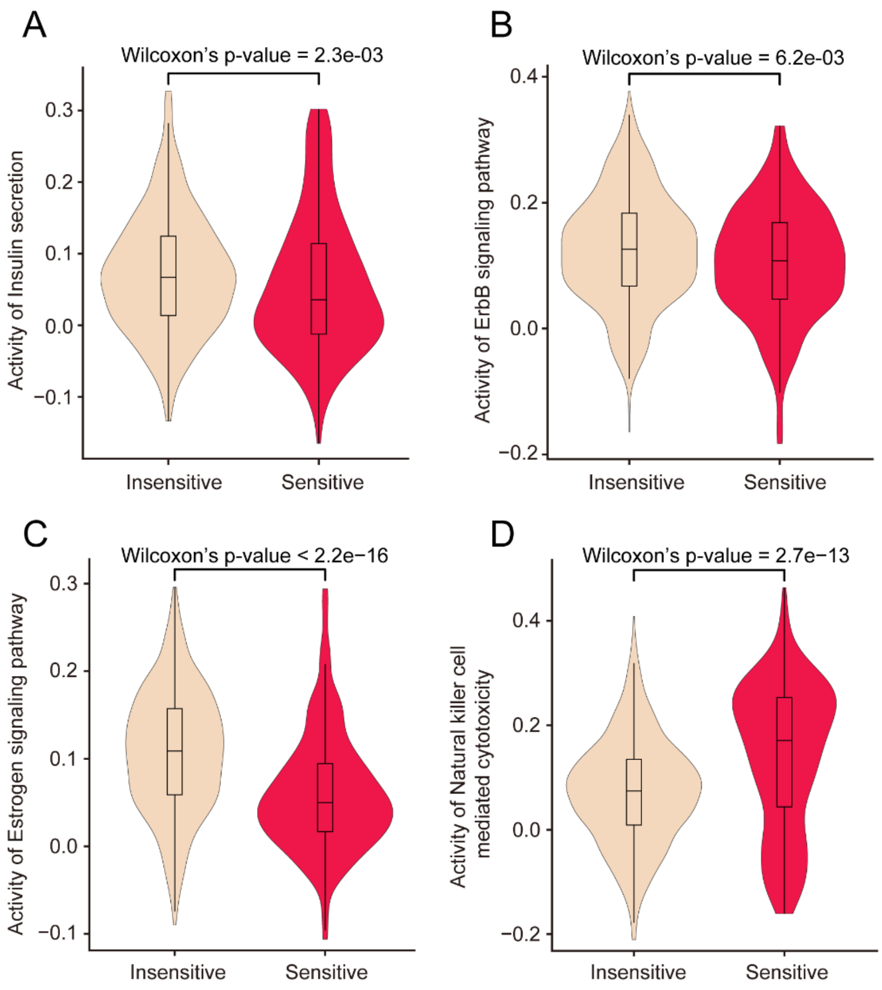

Supplementary Figure S13. (A-D) Violin plots show the relationship between the activity of some important pathways and drug sensitivity. We used a two-sided Wilcoxon rank-sum test to assess the differences between insensitive and sensitive groups.

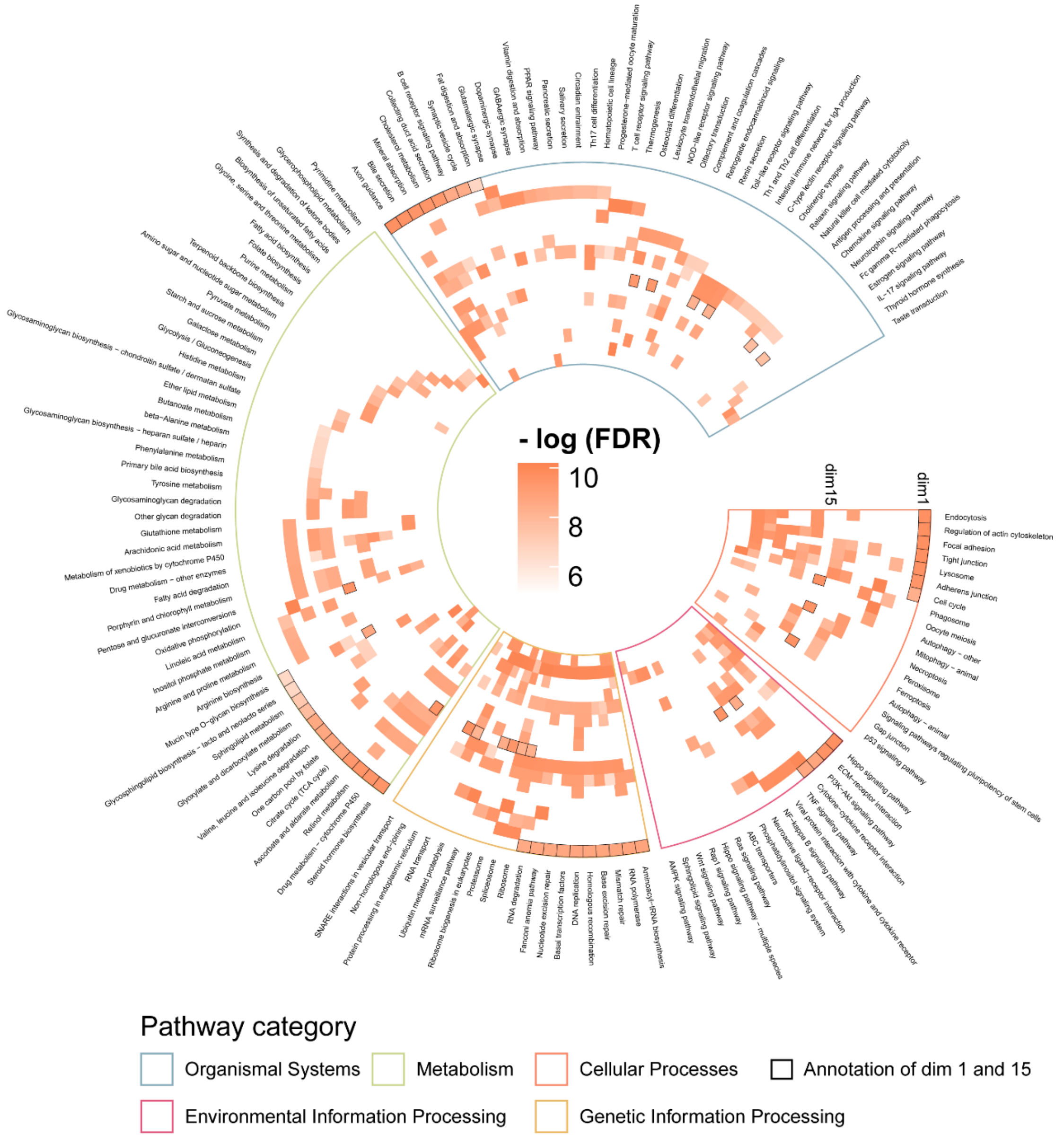

Supplementary Figure S14. Biological annotation of mutation embedding features. Different colored regions represent different annotation pathway categories. Annotations for dimensions 1 and 15 are highlighted with bold borders.

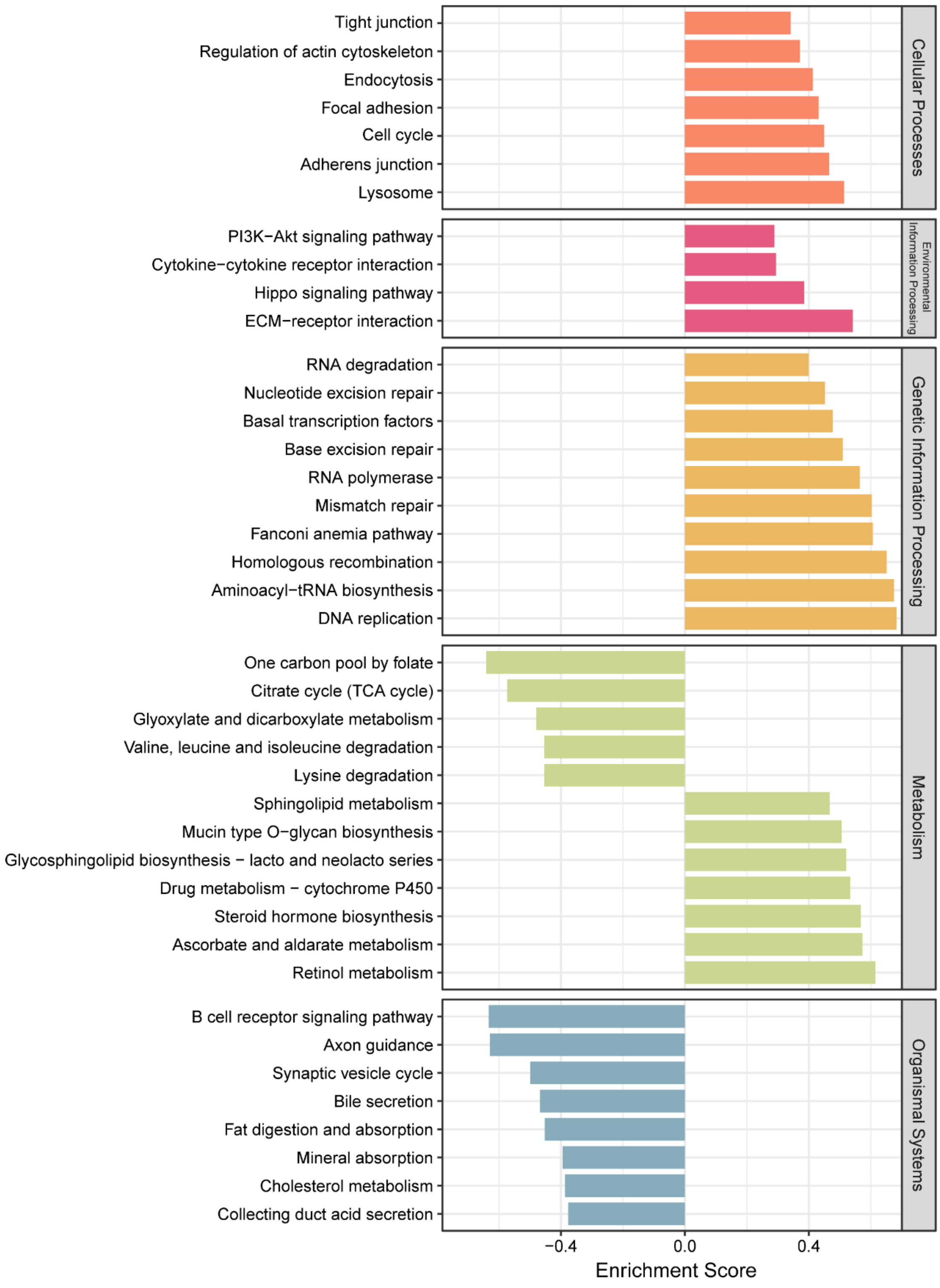

Supplementary Figure S15. Biological annotation of dimension 1. Different colors represent different annotation pathway categories.

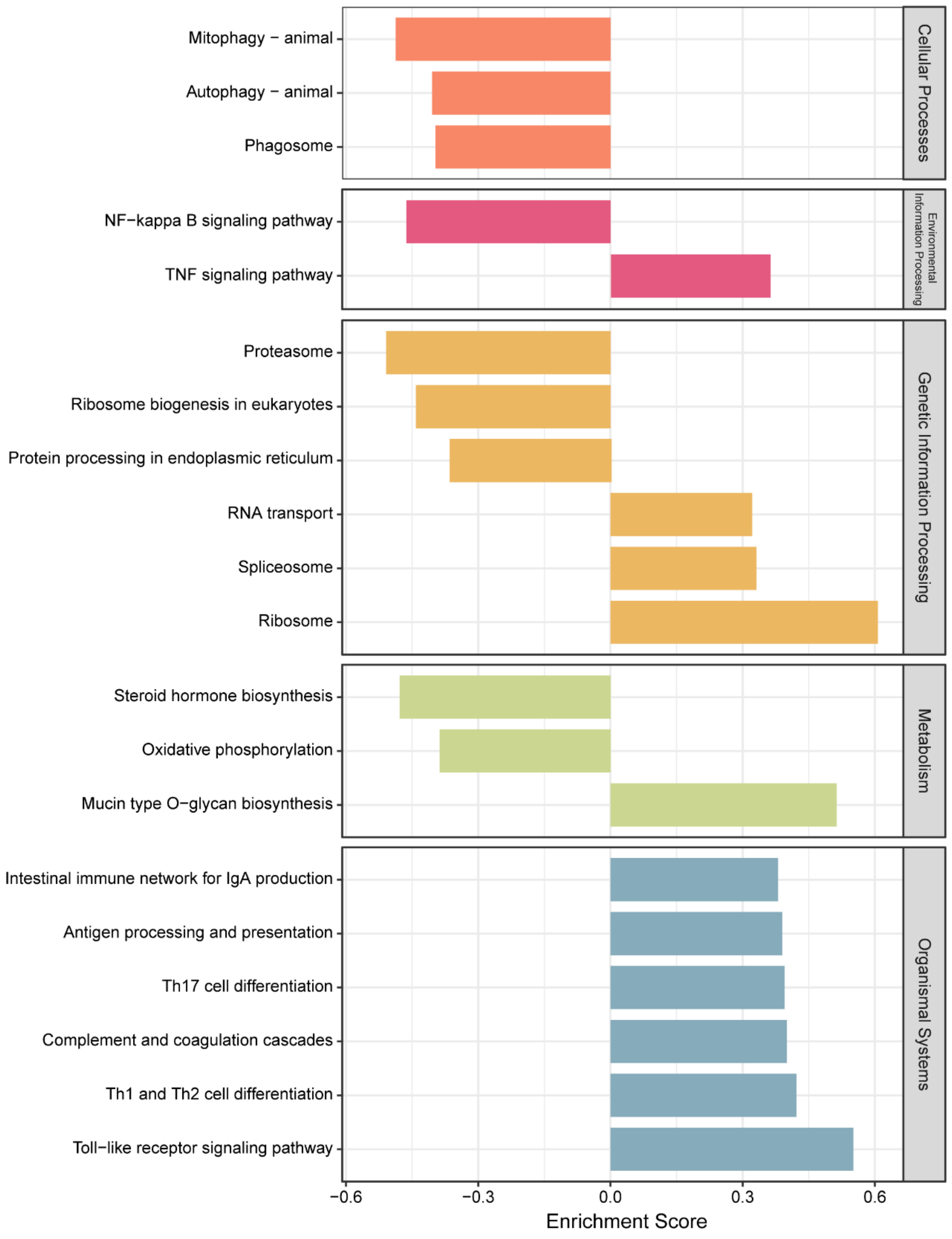

Supplementary Figure S16. Biological annotation of dimension 15. Different colors represent different annotation pathway categories.

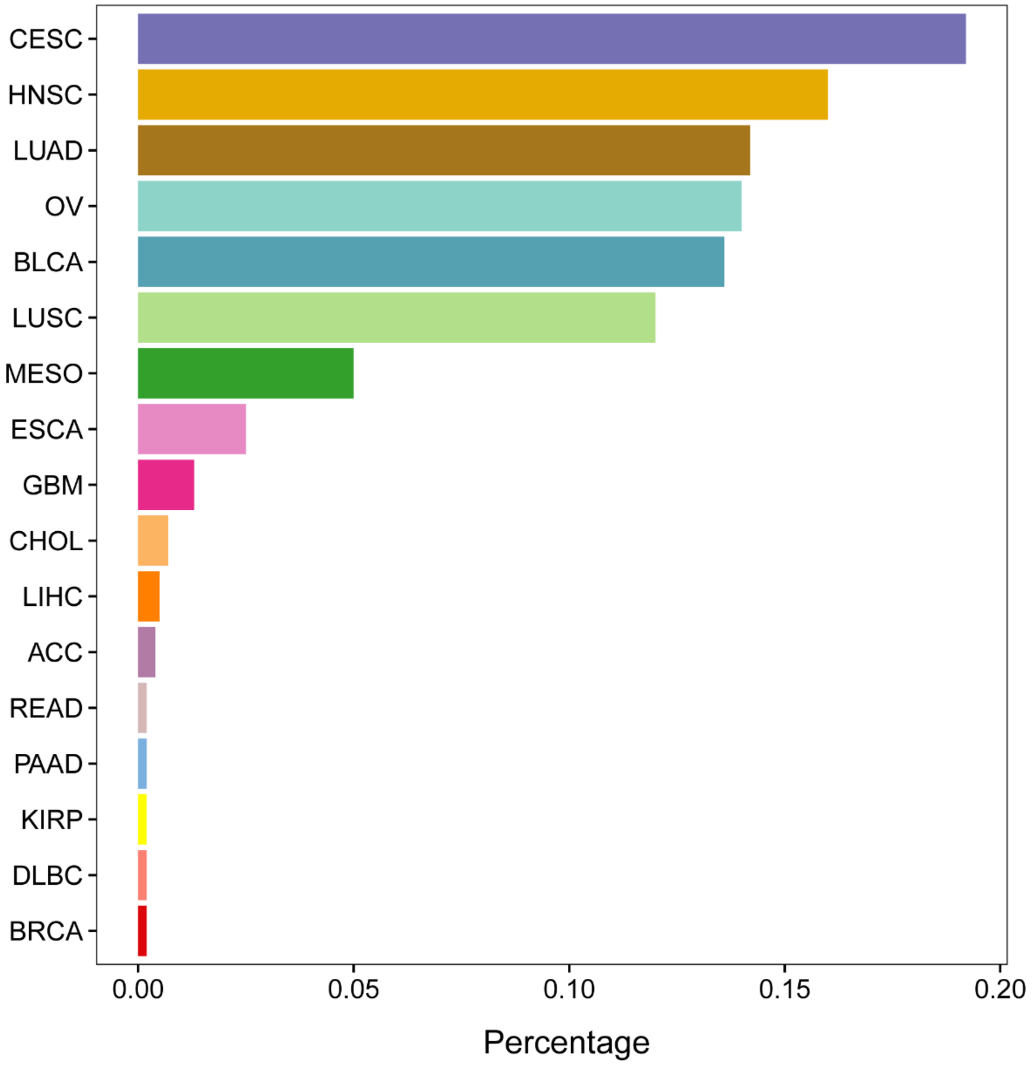

Supplementary Figure S17. The percentage of patients with different cancer types among all patients treated with cisplatin.

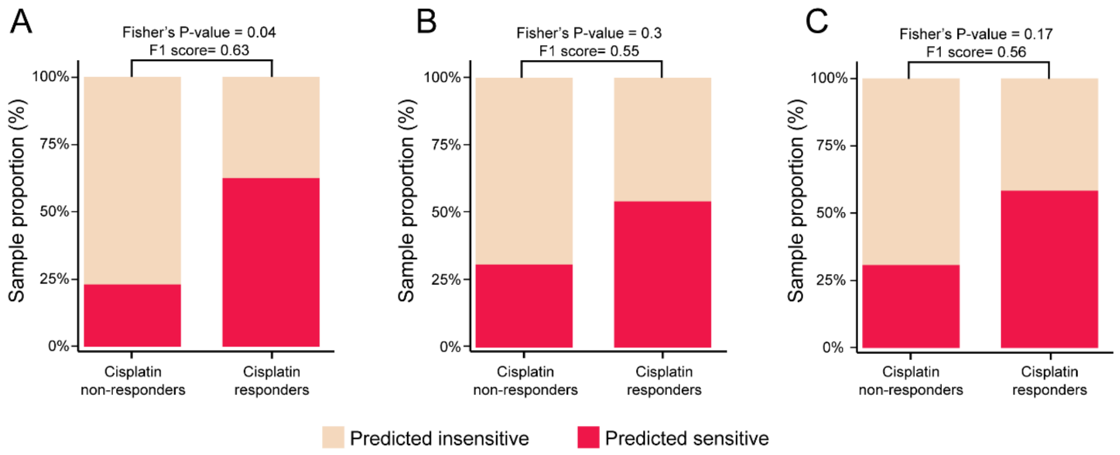

Supplementary Figure S18. Evaluation of the performance of **(A)** DeepCCDS, **(B)** DrugFormer, and **(C)** SpaRx methods in predicting drug responses for BLCA patients through Fisher's exact test and F1 score. The x-axis indicates the actual response status of patients to cisplatin. The stacked bar plot displays the proportion of predicted cisplatin-sensitive patients in the actual responders for cisplatin.

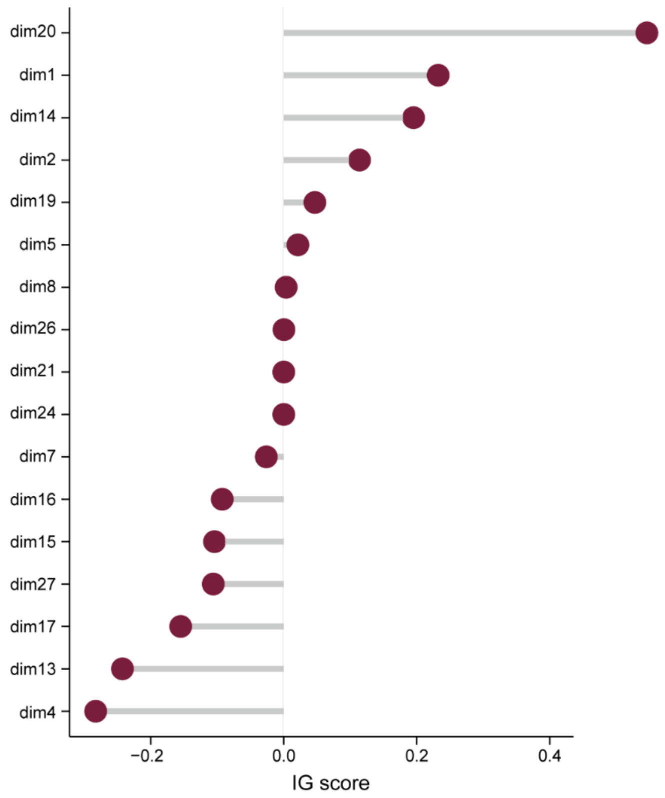

Supplementary Figure S19. Importance (IG score) of mutation embedding features in predicting patient response to cisplatin treatment.

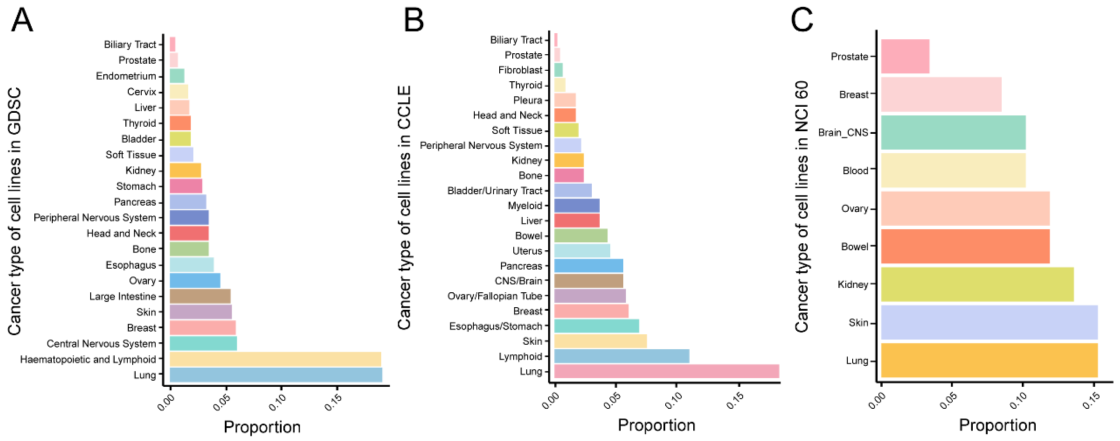

Supplementary Figure S20. The bar plot illustrates the distribution of cancer types for the cell lines used in this study from the **(A)** GDSC, **(B)** CCLE, and **(C)** NCI 60 databases.
